# Bridging the Scales via Personalized Cellular Modeling and Deep Phenotyping in Schizophrenia

**DOI:** 10.1001/jamapsychiatry.2026.0576

**Published:** 2026-03-28

**Authors:** Florian J. Raabe, David Popovic, Clara Vetter, Laura E. Fischer, Genc Hasanaj, Berkhan Karslı, Tim J. Schäfer, Valeria Almeida, Alessia Atella, Miriam Gagliardi, Emanuel Boudriot, Vladislav Yakimov, Lucia Trastulla, Tengjia Jiang, Clara Weyer, Lukas Roell, Joanna Moussiopoulou, Lenka Krčmář, Sabrina Galinski, Irina Papazova, Oliver Pogarell, Alkomiet Hasan, Eva C. Schulte, Andrea Schmitt, Nikolaos Koutsouleris, Anna Levina, Elias Wagner, Moritz J. Rossner, Sergi Papiol, Peter Falkai, Daniel Keeser, Michael J. Ziller

**Affiliations:** 1 Max Planck Institute of Psychiatry, Munich, Germany; 2 Department of Psychiatry and Psychotherapy, University Hospital, Ludwig-Maximilians-Universität (LMU) Munich, Munich, Germany; 3 NeuroImaging Core Unit Munich, University Hospital, LMU Munich, Munich, Germany; 4 Evidence-Based Psychiatry and Psychotherapy, Faculty of Medicine, University of Augsburg, Augsburg, Germany; 5 University of Tübingen, Tübingen, Germany; 6 Max Planck Institute for Biological Cybernetics, Tübingen, Germany; 7 Department of Psychiatry, University of Münster, Münster, Germany; 8 Center for Soft Nanoscience, University of Münster, Münster, Germany; 9 International Max Planck Research School for Translational Psychiatry, Munich, Germany; 10 Systasy Bioscience GmbH, Munich, Germany; 11 Department of Psychiatry, Psychotherapy, and Psychosomatics, Medical Faculty, University of Augsburg, Augsburg, Germany; 12 German Center for Mental Health, partner site Munich-Augsburg, Munich, Germany; 13 Institute of Human Genetics, University Hospital, Faculty of Medicine, University of Bonn, Bonn, Germany; 14 Department of Psychiatry and Psychotherapy, University Hospital, Faculty of Medicine, University of Bonn, Bonn, Germany; 15 Institute of Psychiatric Phenomics and Genomics, University Hospital, LMU Munich, Munich, Germany; 16 Laboratory of Neurosciences, Institute of Psychiatry, University of São Paulo, São Paulo, Brazil; 17 Institute of Psychiatry, Psychology and Neuroscience, King’s College London, London, United Kingdom; 18 Munich Center for Neurosciences, LMU Munich, Planegg-Martinsried, Germany

## Abstract

**Importance:**

While growing evidence implicates synaptic dysfunction as a key pathophysiological mechanism in cognitive impairments in schizophrenia (SCZ), it remains unknown how individual alterations in synaptic connectivity translate into corresponding neural circuit dysfunction and cognitive deficits.

**Objective:**

To test whether genetically driven variability in excitatory neurons’ transcriptome and synapse density in patient-derived neurons in vitro explain individual changes in cortical morphology, electrophysiology, and cognitive impairments in vivo.

**Design, Setting, and Participants:**

This multimodal case-control study integrated deep clinical phenotyping (magnetic resonance imaging, electroencephalography, and cognitive assessments) across 2 independent cohorts with schizophrenia and healthy controls (N = 461) with donor-matched induced pluripotent stem cell (iPSC)–derived neurons (n = 80). Machine learning, transcriptome imputation, and reverse dynamic causal modeling were applied to link cellular and systems-level phenotypes. Data were collected between September 16, 2014, and November 10, 2023, and analyzed from January 2022 to January 2026.

**Main Outcomes and Measures:**

The primary outcome was associations between cellular phenotypes (gene expression, synapse density) and individual-level brain structure, electrophysiology, and cognition.

**Results:**

This multiscale translational framework was implemented in 461 individuals with SCZ and healthy controls across 2 independent cohorts. In both cohorts (cohort 1 [C1]: mean [SD] age: 35.1 [11.6] years; 46 female participants [31.1%]; cohort 2 [C2]: mean [SD] age, 36.9 [11.7] years; 140 female participants [44.57%]), cognitive impairments in SCZ were associated with specific gray matter volume reductions across multiple brain regions, in particular the right dorsolateral prefrontal cortex, as well as disturbed electrophysiological activity in the gamma band. Importantly, the individual-level differences in the genetically driven neuronal gene expression patterns and synapse density in vitro predicted the macro-scale alterations of brain structural (C1: *r* = 0.39; 95% CI, 0.21-0.55; *P* < .001; iPSC: *r* = 0.31; 95% CI, −0.07 to 0.60; *P* = .049; C2: *r* = 0.23; 95% CI, 0.07-0.37; *P* = .003), electrophysiological (theta: *r* = 0.19; 95% CI, 0.04-0.32; *P* = .05; gamma1: *r* = 0.17; 95% CI, 0.028-0.31; *P* = .005; gamma2: *r* = 0.22; 95% CI, 0.07-0.35; *P* < .001), and cognitive (C1: *r* = 0.76; 95% CI, 0.66-0.83; *P* < .001; iPSC: *r* = 0.77; 95% CI, 0.57-0.89; *P* < .001; C2: *r* = 0.17; 95% CI, 0.02-0.32; *P* = .02) phenotypes in vivo, providing a mechanistic link from synapse deficits to cognitive impairments in SCZ.

**Conclusions and Relevance:**

These findings establish a patient-specific link between genetically driven alterations in gene expression, synaptic dysfunction, and large-scale brain and cognitive phenotypes in SCZ. This multiscale framework provides a foundation for mechanism-based stratification and precision target identification for cognitive impairment.

## Key Points

**Question:**

Do synaptic deficits in patient-derived neurons predict individual differences in brain circuitry and cognitive impairment in schizophrenia (SCZ)?

**Finding:**

In a multiscale framework integrating magnetic resonance imaging, electroencephalography, and cognitive data from more than 500 participants with in vitro phenotyping of donor-matched induced pluripotent stem cell (iPSC)–derived neurons, reduced excitatory synapse density and transcriptomic signatures predicted the individual alterations in brain structure, electrophysiology, and cognition in vivo, providing a mechanistic link from synapse deficits to cognitive impairments in SCZ.

**Meaning:**

This study establishes a patient-specific bridge from synapse biology to the individual clinical phenotype, offering a road map for mechanism-based target and drug discovery.

## Teaser

This genetic association study tests whether genetically driven variability in excitatory neurons’ transcriptome and synapse density in patient-derived neurons in vitro explain individual changes in cortical morphology, electrophysiology, and cognitive impairments in vivo.

## Introduction

Converging evidence suggests that cognitive deficits in schizophrenia (SCZ) originate in part from synaptic dysfunction, arising from a combination of genetic and environmentally triggered mechanisms,^1,2,3,4^ which disrupt micro- and macro-connectivity of brain circuits.^1^ Genome-wide association studies (GWAS) have identified dozens of risk genes involved in synaptic function and neurotransmission.^4^ Moreover, meta-analyses consistently demonstrate structural gray matter volume (GMV) reductions across multiple brain regions, including the dorsolateral prefrontal cortex (DLPFC), in individuals with SCZ.^5,6,7,8,9^ Importantly, postmortem and animal studies indicate that GMV changes primarily approximate reductions in dendritic volume and synaptic density.^1,10,11,12^ Conversely, electroencephalography (EEG) studies in SCZ revealed aberrant theta and gamma frequency bands,^13,14^ reflecting disrupted neural synchrony, which is linked to the disorder’s characteristic cognitive deficits in working memory, attention, and executive function.^14^

However, despite insights across multiple scales, these findings have remained largely disconnected, and the association of intermediate phenotypes with underlying neurobiological mechanisms remains elusive.

Personalized disease models–based induced pluripotent stem cells (iPSCs) offer a pivotal technology to dissect disease-relevant molecular mechanisms in SCZ in vitro.^15,16,17^ These models capture the entire (poly)genetic risk profile of their donors and allow assessment of their joint molecular and cellular effects in disease-relevant cell types.^15^ We recently identified alternative polyadenylation as one genetically driven mechanism causing a reduction of synaptic density in iPSC-derived neurons in SCZ, independent of microglial pruning.^18^ Most importantly, iPSCs provide the unique opportunity to link microscale molecular and cellular level changes with macro-scale intermediate phenotypes.^19,20^ Establishing such links would position iPSC technology as a powerful bridge for personalized and target-based interventions in SCZ.^21^

**Figure 1.**
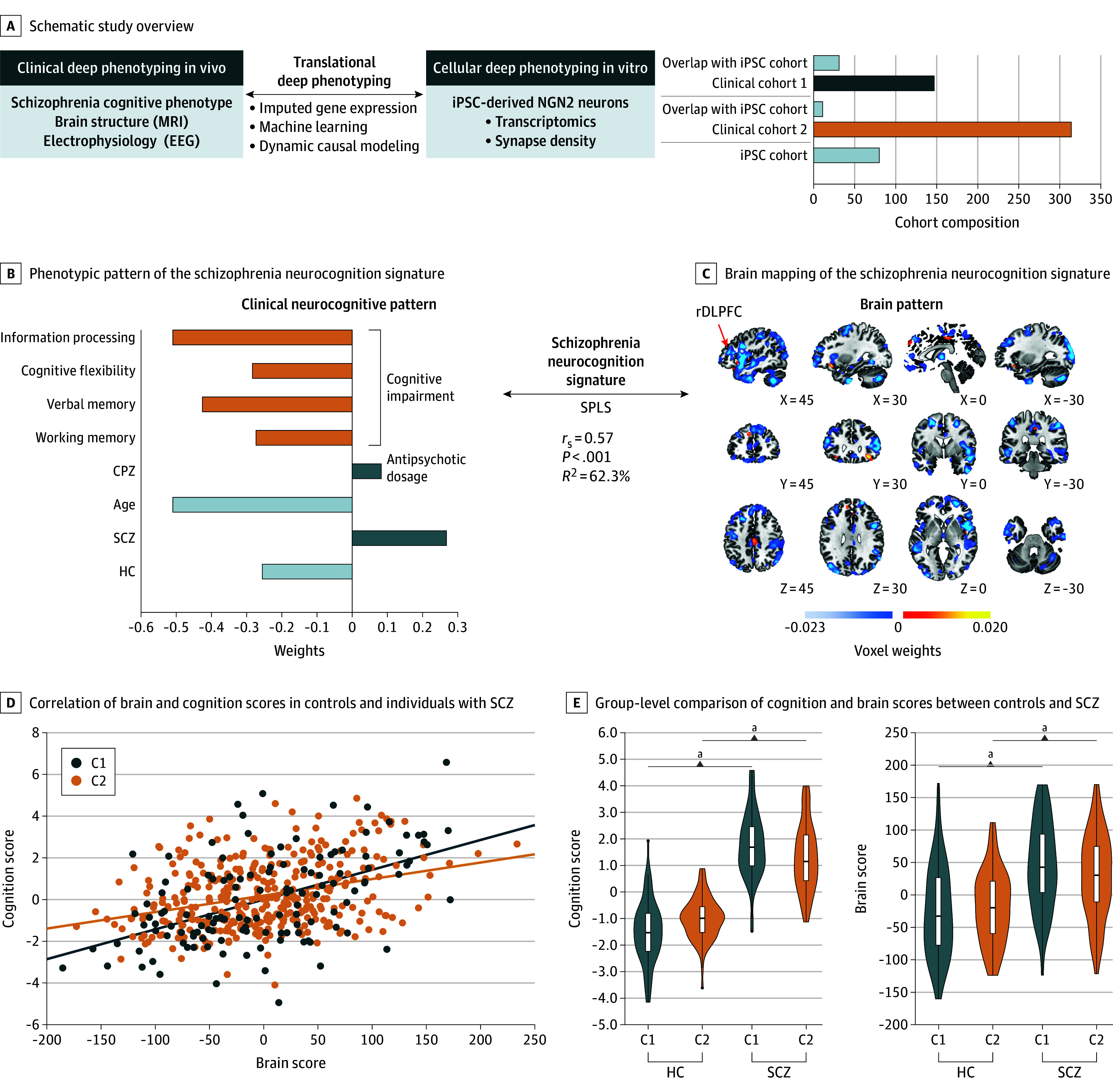
Translational Deep Phenotyping Reveals a Multivariate Phenotype Brain Signature of Cognitive Impairments in Schizophrenia (SCZ) That Is Associated With the Genetic Risk A, Schematic study overview with bar plot illustrates the study design of 2 deep phenotyped cohorts 1 (C1, dark blue) and 2 (C2, orange)^20^ that overlaps with an induced pluripotent stem cell (iPSC) cohort^18^ (light blue) to bridge the translational gap between genetic, molecular, and cellular alterations in vitro and the in vivo individual intermediate phenotype of cognitive impairment in individuals with SCZ. B, Bar plot visualizing the phenotypic pattern of the schizophrenia neurocognition signature captured by the multivariate sparse partial least-squares (SPLS) analysis in the discovery cohort C1. The arrow indicates the Spearman coefficient (*r*) and associated *P* value as well as the coefficient of determination (*R*^2^) between the cognition pattern and the brain pattern within the signature (n = 131; *r* = 0.57; *P* < .001, *t* test; *R*^2^ = 62.3%). C, Brain mapping of the corresponding brain pattern of the schizophrenia neurocognition signature. Positive weighting of voxels in red and negative weighting in blue color scale. D, Correlation of individual brain and cognition scores derived from the applied schizophrenia neurocognition signature on patients with SCZ and healthy controls (HC) in C1 (dark blue, n = 131; *r* = 0.49; *P* < .001, *t* test) and validation cohort C2 (orange, n = 160; *r* = 0.31; *P* < .001, *t* test) with linear regression lines. E, Group-level comparison of individual cognition (left) and brain scores (right) from the schizophrenia neurocognition signature between HC (n_C1_ = 73; n_C2_ = 84) and SCZ (n_C1_ = 58; n_C2_ = 76) individuals in C1 (dark blue) and C2 (orange) cohorts using the Wilcoxon sum rank test (C1: *P* value_brainscore_ = 6.8e-07; *P* value_cognitionscore_ = 1.15e-18; C2: *P* value_brainscore_ = 8.7e-06; *P* value_cognitionscore_ = 1.11e-22). MRI indicates magnetic resonance imaging; NGN2, Neurogenin-2; rDLPFC, right dorsolateral prefrontal cortex. ^a^Indicates *P* < .001.

Here, we present a translational research framework designed to integrate molecular, cellular, and systems-level macrocircuit variability to uncover the neurobiological underpinnings of cognitive impairments in SCZ. Specifically, we combined deep multimodal phenotyping—including magnetic resonance imaging (MRI), EEG, and neurocognitive assessments across 2 independent cohorts^20,22^—with molecularly characterized iPSC-derived neurons from overlapping donors,^18^ machine learning prediction of genetically regulated transcriptomes, and reverse personalized dynamic-causal modeling (rpDCM). This unique multiscale approach enabled us to test whether genetically driven cell-autonomous variability in excitatory neurones and synapses accounts for individual differences in cortical structure, electrophysiology, and cognitive performance (Figure 1A).

## Methods

### Participants and Study Design

This study integrates 2 independent and deeply phenotyped cross-sectional SCZ cohorts^20,22^ (N = 461) with overlapping donors from an iPSC cohort^18^ (n = 80) (Figure 1A), both approved by the the local ethics committee of Faculty of Medicine, Ludwig-Maximilians-Universität (LMU) Munich according to the principles of the Declaration of Helsinki. The included patients with SCZ spectrum disorders and healthy controls (HC), with a subset of unaffected first-degree relatives, provided written informed consent. Cognitive domains were assessed using standardized instruments encompassing verbal memory, working memory, information processing, cognitive flexibility, and crystalline intelligence.^23^ Antipsychotic doses were harmonized using chlorpromazine equivalents (CPZeq^24^; details in the eMethods in Supplement 1). Study C2 was registered in the German Clinical Trials Register (DRKS, identifier DRKS00024177).

### MRI and EEG

Structural T1-weighted images were acquired from 3T MRI scans and processed using the CAT12 toolbox. GMV was analyzed on a whole-brain voxelwise (2-mm^3^ voxel size) basis. Resting-state EEG (rsEEG) was recorded for 10 minutes using a 32-channel system with Cz as the reference electrode. Power spectral density (PSD) features were computed to extract absolute and relative power^25^ of the different frequency bands: delta (1-4 Hz), theta (4-8 Hz), alpha (8-12 Hz), beta (12-30 Hz), gamma1 (30-50 Hz), and gamma2 (50-70 Hz). For further details, see the eMethods in Supplement 1.

### iPSC-Derived Neurons

Induced differentiation of iPSC-derived excitatory pyramidal neurons (iNs) from the 80 donors included for this study was carried out as described previously.^18^ For details of neuronal differentiation, technical quality control analysis, RNA-Seq data processing and analysis, imaging, and synapse density analysis, see the eMethods in Supplement 1 and supplements of our companion study.^18^

### Polygenic Risk Scores and Imputed Gene Expression

To calculate SCZ polygenic risk scores (SCZ-PRS) based on PGC3 GWAS,^4^ single-nucleotide polymorphism (SNP) effect sizes were estimated using PRS–continuous shrinkage (PRS-CS).^26^ Based on the recently released CASTom-iGEx approach^27^ combined with support vector regression (SVR), we predicted gene expression levels in iNs from genotype in cis and in trans (eMethods in Supplement 1).

### Reverse Personalized Dynamic Causal Modeling

We adapted a previously established canonical microcircuit Dynamic Causal Modeling (DCM) model^28^ to develop a reverse personalized DCM (rpDCM) to link patient-specific in vitro synaptic density data with in vivo rsEEG signatures. Canonical microcircuit parameters were customized using empirically measured synapse data from iNs to simulate rsEEG-PSD via rpDCM. Implementation details, parameter mapping, and validation procedures are described in the eMethods in Supplement 1.

### Statistical Analysis

Multilayered associations between clinical and cognitive phenotype and GMV were identified using the sparse partial least-squares (SPLS) toolbox by Popovic and colleagues.^29,30,31^ SPLS uses singular-value decomposition to extract latent variables ( = multivariate signatures) that capture the shared covariance between 2 matrices—in our case, the cognitive phenotype matrix and the GMV matrix. By projecting each individual’s data onto the weight vectors of the latent variable (eg, schizophrenia neurocognition signature), individualized latent scores (eg, cognition score and brain score) can be derived, providing individual loadings of these biobehavioral signatures that can be used for further downstream analyses (eMethods in Supplement 1).

Associations between the transcriptomic signatures, individualized cognition scores, brain score, and electrophysiological phenotypes were assessed using linear SVR models within the NeuroMiner platform.^32,33^ Group comparisons and association analyses were performed using linear (mixed) or logistic regression models as indicated in the respective sections. Multiple comparisons correction was done where appropriate using the false discovery rate (FDR) method (eMethods in Supplement 1).

All analyses were conducted using MATLAB R2022a (MathWorks) and R (R Foundation); specifically, functions from the MATLAB Statistics and Machine Learning Toolbox, as well as the machine learning platform NeuroMiner, release version 1.3, were used. Two-tailed *P* < .05 was considered significant.

## Results

### Structural Brain Alterations Linked to Cognitive Impairment in SCZ

To determine the behavioral consequences of changes in region-specific synaptic density, we considered alterations in GMV as approximate measure.^1,10,11^ Based on previous evidence indicating structural brain alterations in SCZ,^2,6,34^ we hypothesized that region-specific changes in GMV might particularly contribute to cognitive impairment as one critical clinical phenotype. To test this hypothesis, we sought to identify a multimodal signature of cognitive alterations in SCZ and its link to region-specific structural GMV brain changes in 2 deeply phenotyped cohorts (cohort 1 [C1]: n = 147; mean [SD] age, 35.1 [11.6] years; 46 female participants [31.1%]; cohort 2 [C2]: n = 314; mean [SD] age, 36.9 [11.7] years; 140 female participants [44.57%]) (Figure 1A; eTable 1 in Supplement 2). Within the discovery cohort (C1), the SPLS algorithm^29^ identified 2 significant signatures, each representing a distinct combination of phenotypic (cognitive and clinical parameters) and structural GMV brain patterns.

In line with the literature,^35,36^ the first aging signature represented a brain pattern of widespread GMV reduction (Spearman ρ = 0.78; *P* < .001; n = 131) (eFigure 1A in Supplement 1; eTables 2-5 in Supplement 2).

The second schizophrenia neurocognition signature (Spearman ρ = 0.57; *P* < .001; n = 131), which was independent of the age effect already captured by the aging signature, identified individuals with SCZ (positive weight; negative weight for HC) encompassing pronounced cognitive impairments in information processing, verbal long-term memory, cognitive flexibility, and working memory (indicated by negative weighting), as well as a higher disease severity (negative weight for age, positive weight for CPZeq) (Figure 1B; eFigure 1B in Supplement 1). This neurocognitive pattern was linked to a brain pattern of mainly GMV reductions in the frontotemporal regions, as well as the right insula, right DLPFC (rDLPFC), amygdala, and hippocampus and mapped predominantly to the default, limbic, control, and salience networks (Figure 1C; eFigure 1C in Supplement 1; eTables 2-5 in Supplement 2). Importantly, this signature was independent of sex.

By projecting the participants’ data of validation cohort C2 onto the SPLS signature that was trained independently in C1, we could reveal that the cognition and brain scores were also significantly correlated in C2. Thereby, we successfully confirmed the generalizability of the schizophrenia neurocognition signature (Figure 1D), despite different study protocols regarding the applied cognitive tasks, as well as the MRI scanners.

Importantly, the latent brain and cognition scores—the individual loadings onto the schizophrenia neurocognition signature—were significantly higher in SCZ (Figure 1E). Finally, higher cognition (dysfunction) scores and brain scores were both linked to lower occupational rates, and higher cognition scores were associated with lower Global Assessment of Functioning scores (eFigure 2 in Supplement 1).

In summary, the SPLS analysis identified a schizophrenia neurocognition signature that captures a reproducible profile of severe cognitive impairments linked to a pattern of GMV loss within macrocircuits critical for cognitive function.^8,37,38^

### Patient-Level Neuronal Transcriptomes Associate With Brain Intermediate Phenotypes

Significant correlation of both scores with SCZ-PRS^4^ indicated that the schizophrenia neurocognition signature captures trait-specific aspects of the illness within our cohorts of European ancestry (eFigure 3, eFigure 4A in Supplement 1). Of note, the cognition score showed a stronger association with SCZ-PRS than the brain score, suggesting that the clinical neurocognitive pattern of the schizophrenia neurocognition signature is closer associated to the trait-driven variability compared to its brain pattern.

Based on these findings and our previous iPSC study that identified molecular mechanisms of synapse dysfunction in SCZ,^18^ we hypothesized that genetically driven alterations in neuron-related gene expression patterns contribute to the emergence of this signature. We therefore used the established iPSC cohort^18^ (n = 80) of patients with SCZ and HCs that overlap with a subset of the translational cohorts C1 and C2 (Figure 1A).^20^ Previously, we described the cohort-level in vitro differentiation into iNs (eFigure 4B in Supplement 1), giving rise to a deeply phenotyped personalized disease model library, including whole transcriptome profiles.^18^ iN differentiation was performed in replicated batches across 2 centers to account for technical variability.^18^ Importantly, extensive quality control of the iPSCs and the differentiated iNs showed no difference between case-control cell culture conditions, maturation state, or the general health of the iN cultures (eFigure 4C, eMethods in Supplement 1; see also our companion study^18^).

First, we leveraged the concept of transcriptome imputation,^39^ predicting the gene expression pattern in iNs from the individual-level genotype using machine learning^27^ in both clinical cohorts (eFigure 4D and E in Supplement 1). Importantly, differential expressed gene (DEG) patterns between SCZ and HC in iNs between predicted and the 310 empirical measured DEGs (ΔSCZ-HC) were correlated (*r* = 0.45; *P* value < 2.2e-16, *F* test) with 75% of nominally significant genes exhibiting concordant direction of effect (eFigure 4F-H in Supplement 1; eTable 6 in Supplement 2).

**Figure 2.**
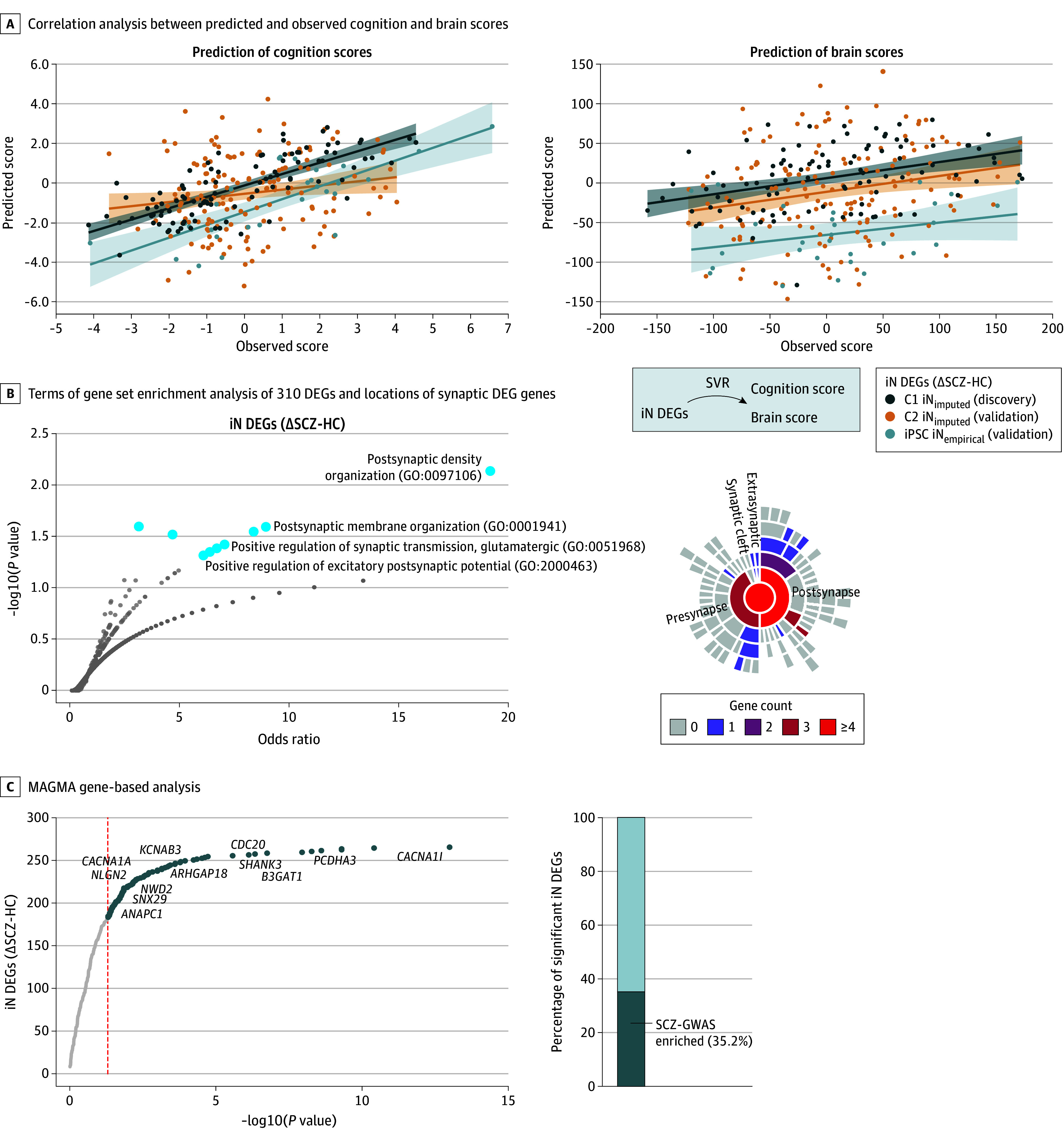
Human Induced Pluripotent Stem Cell (iPSC)–Based Neuronal Transcriptome Predicts Individual Cognition and Brain Scores A, Correlation analysis between the predicted (y-axis) and observed (x-axis) cognition (left) and brain scores (right) of the schizophrenia neurocognition signature based on support vector regression (SVR) using imputed gene expression levels of 310 differential expressed genes (DEGs) (∆SCZ-HC) in iNs in discovery cohort 1 (C1 iN_imputed_, dark blue) and separate validation cohort 2 (C2 iN_imputed_, orange) and held-out empirically measured (not imputed) iN transcriptome data of the iPSC cohort (iPSC iN_empirical_, light blue, deviating offset for visibility). Lines indicate linear regression model for all 3 cohorts for the cognition (n_C1_ = 98; *r*_C1_ = 0.76; p_C1_ = 0.0002; n_C2_ = 153; *r*_C2_ = 0.17; p_C2_ = 0.016; N_iN_ = 29; r_iN_ = 0.77; p_iN_ = 0.0002, permutation test) and brain scores (n_C1_ = 98; *r*_C1_ = 0.39; p_C1_ = 0.0002; n_C2_ = 153; *r*_C2_ = 0.23; p_C2_ = 0.0026; n_iN_ = 29; *r*_iN_ = 0.31; p_iN_ = 0.049, permutation test). B, Left: gene set enrichment analysis of the 310 DEGs (∆SCZ-HC) in iNs (eFigure 4 in Supplement 1; background gene set: iN transcriptome) using Enrichr.^40,41,42,43^ Points represent gene sets with annotations for selected Gene ontology (GO) terms. Larger blue points denote significant GO terms (*P* < .05, not FDR corrected). Right: sunburst plot illustrating locations of synaptic DEG genes based on Synaptic Gene Ontologies.^44^ C, Significance of gene-based association from MAGMA analysis of SCZ genome-wide association study (GWAS) summary statistics^4^ plotted against the ranked list of iN DEGs (SCZ-HC). Significant genes are visualized in blue, selected genes are annotated. Bar plot visualizing percentage of iN DEGs significantly associated with SCZ GWAS^4^ (dark blue).

Next, we explored whether the molecular variability in iN DEGs relates to the macro-scale intermediate phenotypes of the schizophrenia cognition signature (eFigure 5A in Supplement 1). We predicted the individual brain and cognition scores with the imputed DEG profiles of patients with SCZ and HC in C1 using SVR models in a repeated nested cross-validation pipeline, excluding individuals with empirically measured transcriptome profiles. This approach revealed a predictive capacity of the imputed iN DEGs to predict the individual cognition (*r* = 0.76; 95% CI, 0.66-0.83; *P* < .001; area under the curve [AUC] = 0.91) and brain scores (*r* = 0.39; 95% CI, 0.21-0.55; *P* < .001; AUC = 0.69) in the discovery cohort C1 (dark blue in Figure 2A; eFigure 5B in Supplement 1; eTables 7-9 in Supplement 2).^40,41,42,43,44^

Application of the trained SVR model to a held-out patient sample with empirically measured DEGs rather than imputed transcriptome profiles confirmed the predictive performance (light blue in Figure 2A; eFigure 5B in Supplement 1; cognition score: *r* = 0.77; 95% CI, 0.57-0.89; *P* < .001; AUC = 0.95; brain score: *r* = 0.31; 95% CI, −0.07 to 0.60; *P* = .049; AUC = 0.60). Similarly, replication analyses in the validation cohort C2 confirmed the generalizability of the iNs transcriptome-based prediction of the cognition (*r* = 0.17; 95% CI, 0.028-0.31; *P* = .02; AUC = 0.63) and the brain score (*r* = 0.23; 95% CI, 0.07-0.37; *P* = .003; AUC = 0.59), respectively (orange in Figure 2A; eFigure 5B in Supplement 1). Notably, these results were specific to the empirically determined iN DEG feature set, as both the imputed and the empirical DEG iN datasets significantly outperformed 100 models trained on randomized sets of iN genes and our previously published model^27^ using imputed (postmortem) DLPFC gene expression (eFigure 5C in Supplement 1; eTables 10 and 11 in Supplement 2).

Interestingly, the iN DEGs were significantly enriched for synapse-related biological processes, and MAGMA gene-based analyses revealed direct associations with the SCZ GWAS for a substantial part (Figure 2B-C; eTables 12 and 13 in Supplement 2). Moreover, annotation-based analysis of the predictive gene features revealed the contribution of a substantial fraction of the 310 DEGs (ΔSCZ-HC) to the prediction of the individual cognition and brain score (eFigure 5D in Supplement 1) including several neurotransmission-related genes, such as the calcium voltage-gated channel subunit alpha1 A (*CACNA1A*) or the postsynaptic protein dishevelled binding antagonist of beta catenin 1 (*DACT1*) previously associated with neuronal and cognitive function.^45,46^

Jointly, these findings demonstrate that the variability of imputed and empirical measured molecular features from personalized iPSC-based disease models can partially explain the variability of SCZ-related phenotypes. Moreover, they implicate alterations in synaptic gene expression as an important contributor to cognitive impairment. Jointly, these findings support the hypothesis that regional GMV reductions arise in part from synaptic alterations.^2^

### Neurophysiological Consequences of Altered Brain Structure

**Figure 3.**
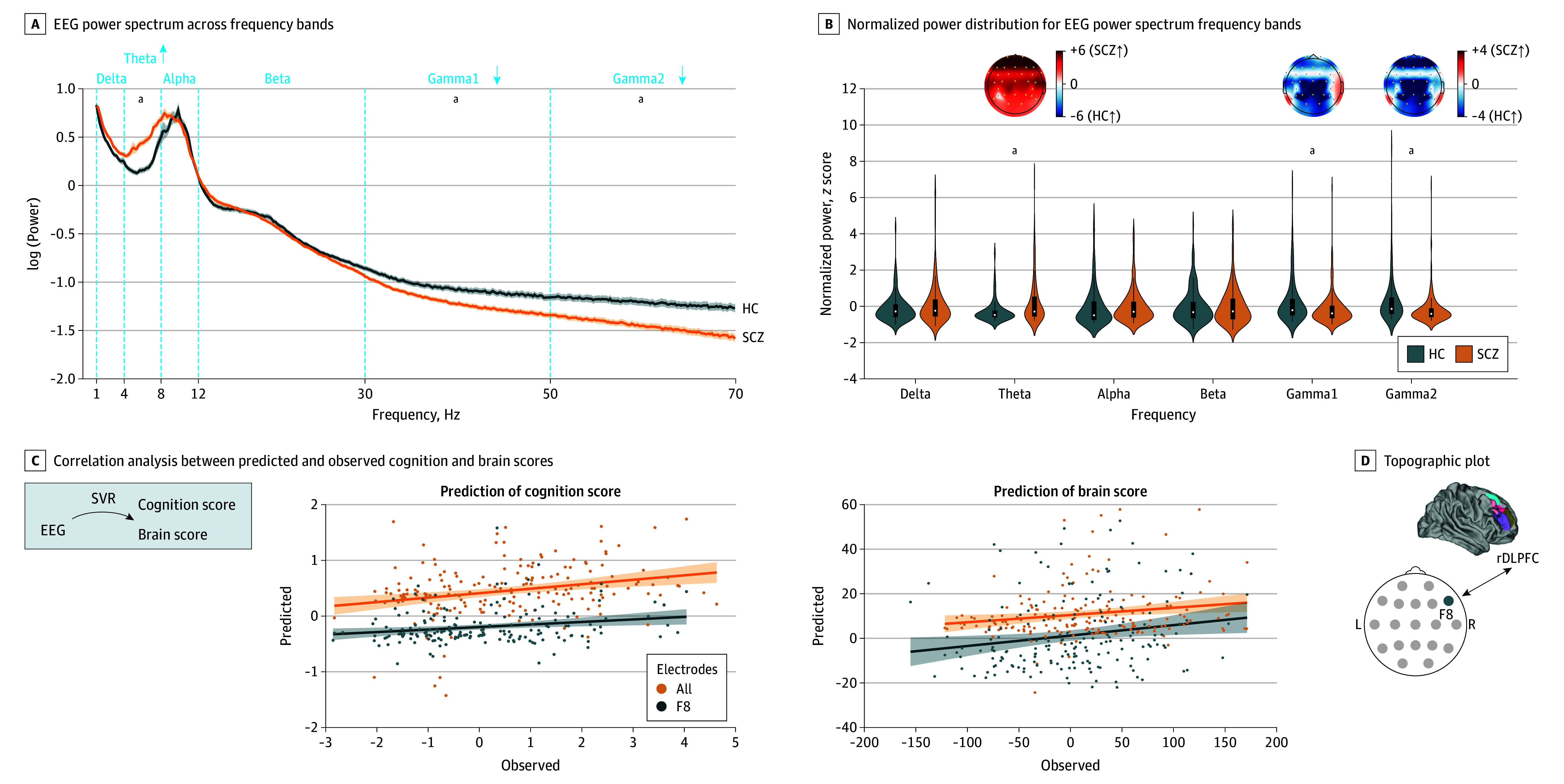
Electroencephalography (EEG) Power Spectrum Alterations in Schizophrenia (SCZ) Relate to Brain Structural and Neurocognitive Changes A, Resting-state EEG power spectrum (1-70 Hz, eyes closed) from 139 healthy controls (HC, blue line) and 217 patients with SCZ (orange line) across analyzed frequency bands delta, theta, alpha, beta, gamma1, and gamma2. Arrows and footnote indicate significant differences in SCZ compared to HC by Mann-Whitney *U* test (*P*[false discovery rate (FDR)] < .001; *U*_delta_ = 1.43621, *U*_theta_ = 4.12, *U*_alpha_ = 1.98, *U*_beta_ = 0.09, *U*_gamma1_ = −3.89, *U*_gamma2_ = −5.97). B, Violin plots depicting the normalized power (*z* scores) distribution for each resting-state EEG power spectrum frequency band in 139 HCs (blue) and 217 patients with SCZ (orange). Group comparison evaluated with Mann-Whitney *U* test (*U*_delta_ = 1.43621, *U*_theta_ = 4.12, *U*_alpha_ = 1.98, *U*_beta_ = 0.09, *U*_gamma1_ = −3.89, *U*_gamma2_ = −5.97). Topoplots illustrate alterations of the spatial EEG power distribution across the scalp in SCZ (heightened in red and diminished in blue). C, Correlation analysis between the predicted (y-axis) and observed (x-axis) cognition and brain scores of the schizophrenia neurocognition signature based on a nested cross-validated support vector regression (SVR) predicting the latter scores from individual EEG frequency band power level in the theta, gamma1, and gamma2 frequency range across all electrodes in the joint C1 and C2 cohorts (orange). Predicted values result from predictions where the respective individual was held out in the outer fold of the nested cross-validation. Lines indicate linear regression between predicted and observed cognition (n = 191; *r* = 0.2; *P* < .001, permutation test) and brain score (n = 191; *r* = 0.17; *P* = .02, permutation test). In addition, SVR-based predictions only for the F8 electrode (blue) are shown (cognition score: n = 191; *r* = 0.36; *P* < .001, permutation test; brain score: n = 191; *r* = 0.28; *P* < .001, permutation test). D, Topo plot indicates the anatomical location of the F8 electrode as the top-ranking predictive feature with highest sum of cross-validation ratio overall mean in the cognition and brain score SVR analysis that reflects electrophysiological activity of the right dorsolateral prefrontal cortex (rDLPFC). ^a^Significant differences in SCZ compared to HC by Mann-Whitney *U* test (*P*[FDR] < .001).

To test the hypothesis that changes in neuronal activity accompany alterations in GMV, we evaluated the link between the schizophrenia neurocognition signature and the brain’s electrophysiological rsEEG (eyes closed) activity pattern, reflecting the aggregated postsynaptic potential.^47^ In line with previous findings,^48^ rsEEG-PSD analysis of available rsEEGs across C1 and C2 revealed an increase in theta and a decrease in gamma1 and gamma2 power in patients with SCZ (Figure 3A-B; eTable 14 in Supplement 2).

SVR-based prediction of the individual brain and cognition scores of the schizophrenia neurocognition signature from the matching patients’ rsEEG-PSD revealed an association between neurophysiology and brain structure (*r* = 0.17; *P* = .01) and cognition (*r* = 0.2; *P* = .004) (Figure 3C). Interestingly, this link was largely driven by the variation in the theta and gamma range. Spatially, these changes were localized to the neighbored electrodes F8, F4, and T4, with F8 being the most predictive feature (Figure 3D; eTables 15-20 in Supplement 2) capturing rDLPFC brain activity, which is strongly involved in executive function and working memory.^49^

These findings on the electrophysiological level are consistent with the strong regional loadings of the schizophrenia neurocognition signature in the rDLPFC, which shows prominent GMV reductions that correlate with cognitive impairment (eFigure 6 in Supplement 1).

**Figure 4.**
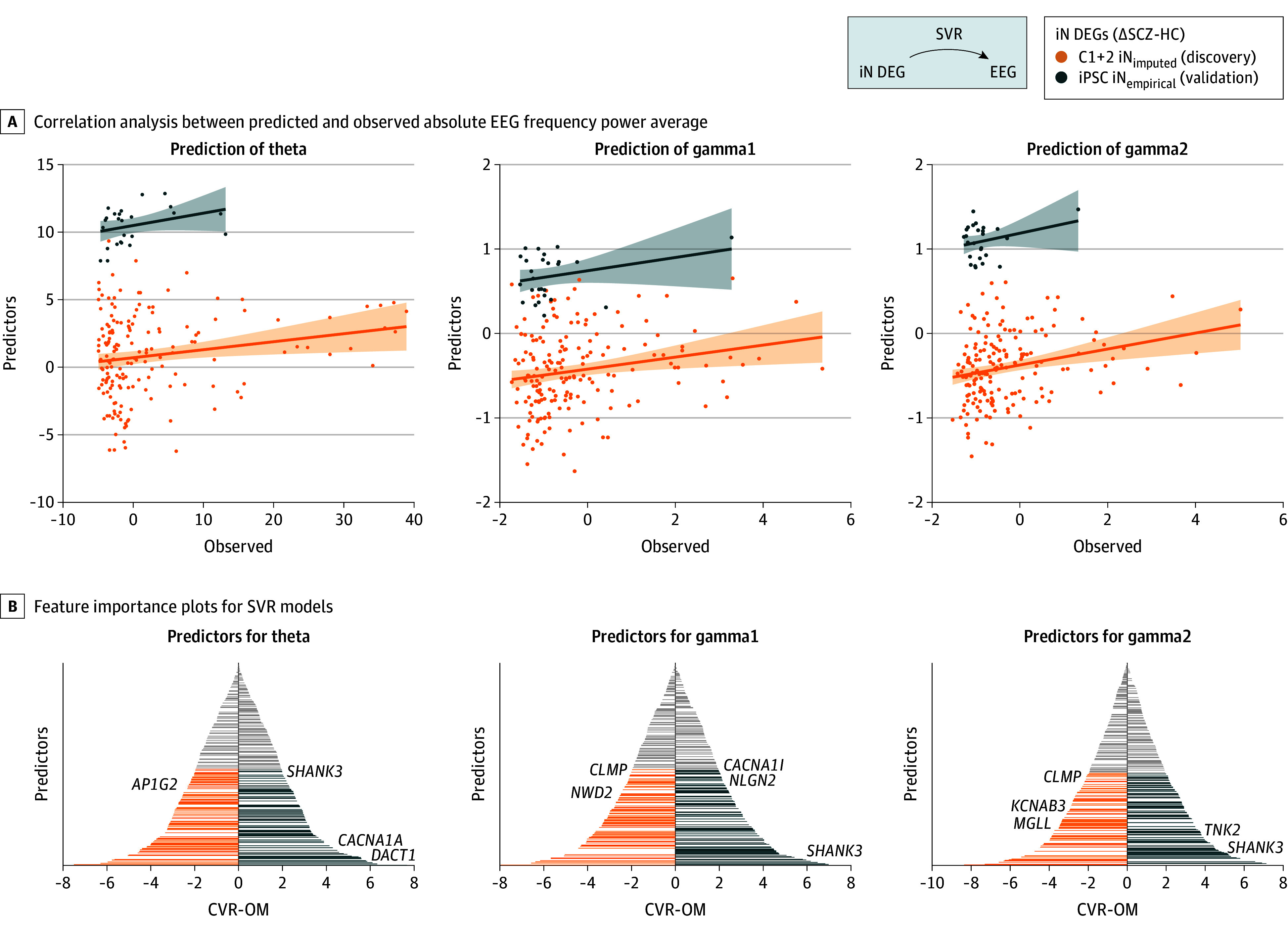
Alterations in Synapse-Related Gene Expression Are Linked to Electroencephalography (EEG) Power Spectrum Alterations A, Correlation analysis between the individual predicted (y-axis) and observed (x-axis) absolute EEG frequency power average over all electrodes in the theta (left), gamma1 (middle), and gamma2 (right) band. Prediction of the band power was performed using support vector regression (SVR) from genotype imputed gene expression levels of 310 induced pluripotent stem cell (iPSC)–derived excitatory pyramidal neuron (iN) differential expressed gene (DEGs) (∆schizophrenia–healthy controls [∆SCZ-HC]) in a fused subset of individuals from cohort 1 (C1) and cohort 2 (C2) with available EEG data (C1 + 2 iN DEG_imputed_; n = 178; discovery, orange). Replication sample from a subset of held-out individuals of the iPSC cohort with empirically measured iN DEGs (iPSC iN DEG_empirical_; n = 26; validation, blue). The SVR model was trained using 5-fold cross-validation, and all predicted values are based on the held-out sample subsets. Lines indicate linear regression models between the observed and predicted values for the discovery cohort and replication sample of theta (*r*_D_ = 0.185; p_D_[permutation test] = 0.006; *r*_iN_ = 0.26; p_iN_[permutation test] = 0.1), gamma1 (*r*_D_ = 0.174; p_D_[permutation test] = 0.01; *r*_iN_ = 0.28; p_iN_[permutation test] = 0.0788; p_iN_[mean average error] = 0.0346), and gamma2 average band power (*r*_D_ = 0.22; p_D_[permutation test] = 0.001; *r*_iN_ = 0.28; p_iN_[permutation test] = 0.08; p_iN_[mean average error] = 0.001), showing the replication iPSC sample with a different offset for better visibility. B, Feature importance plots for the SVR models depicting the ranked cross-validation ratio overall means ([CVR-OM], x-axis) of all gene features (y-axis) in the SVR model to predict the frequency band power for theta (left), gamma1 (middle), and gamma2 (right). Significant features are colored, with annotations for selected genes of interest.

To further connect the macro-scale electrophysiological changes to their prospective molecular basis, we performed SVR-based prediction of SCZ-associated rsEEG-PSD (theta, gamma1, and gamma2) using iN DEG (ΔSCZ-HC) imputed transcriptomic data. This analysis uncovered a significant association of the genetically determined imputed gene expression patterns in iNs with individual theta, gamma1, and gamma2 rsEEG-PSD variability (Figure 4A, in orange; range of theta, gamma1, and gamma2 predictions: *r*_D_ = 0.17-0.22; *P* values range, <.001-.01, permutation test; for details see Figure legend; eTables 21-26 in Supplement 2). Application of these models onto the empirically measured iN transcriptomes within the iPSC cohort replicated the prediction of the individual gamma1 and gamma2 rsEEG-PSD (Figure 4A, blue). Moreover, the link between DEG variability and gamma1 EEG band power was also evident at the level of the F8 electrode, which reflects rDLPFC’s neuronal activity (eFigure 7 in Supplement 1). Importantly, a substantial part of the imputed iN DEGs (ΔSCZ-HC) were predictive of the individual rsEEG-PSD variability, including several key synaptic genes, such as *SHANK3* or *NLGN2* (Figure 4B).

Jointly, these analyses link interindividual variability in molecular processes, contributing to synaptic function and brain structure,^1,10,11^ to changes in patient-level electrophysiology.

### Individual Synaptic Density Associates With Patients’ Electrophysiology

**Figure 5.**
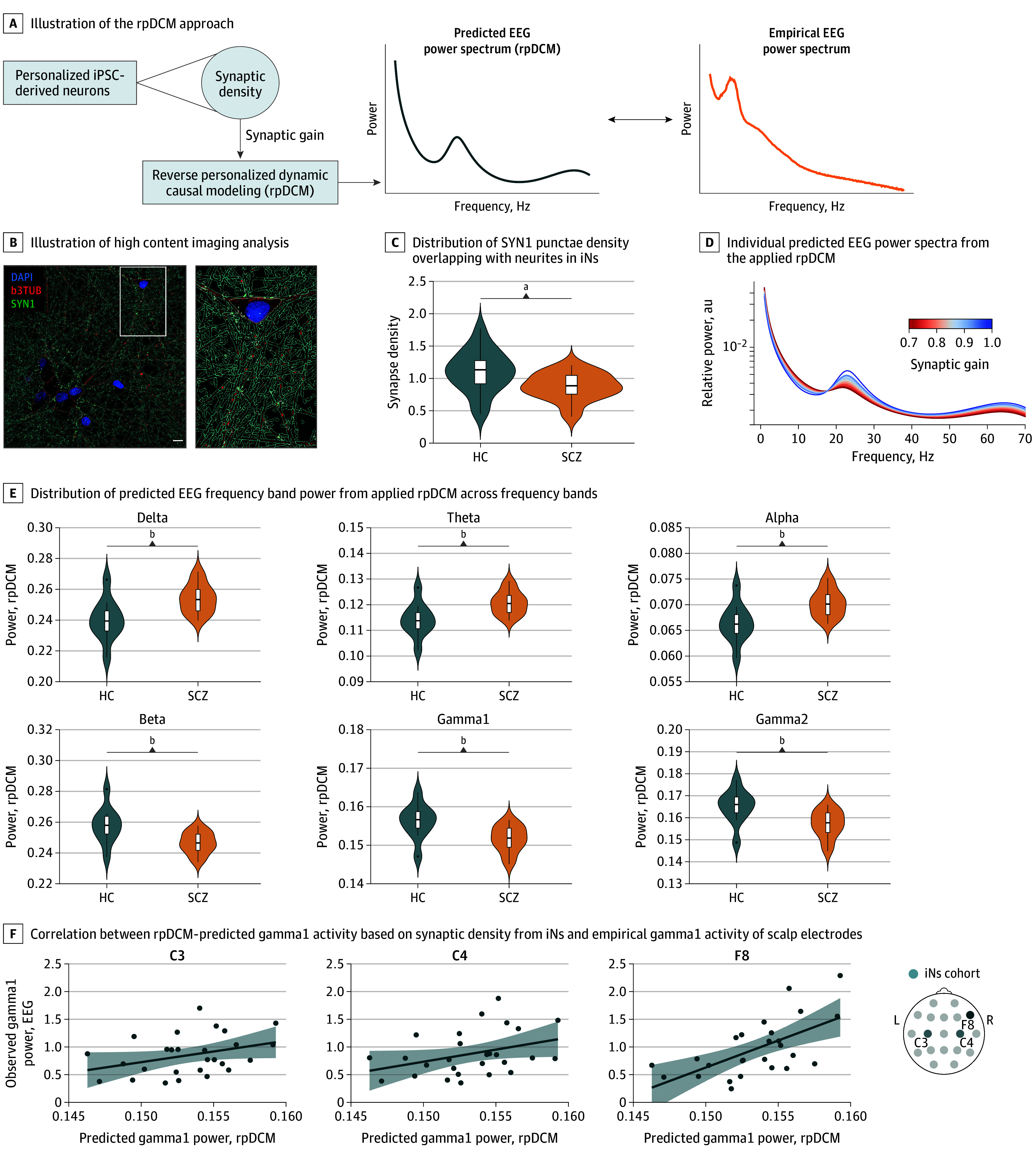
Reverse Personalized Dynamic Causal Modeling (rpDCM) Predicts Individual Electroencephalography (EEG) Gamma Power Based on Intraindividual Synaptic Density From Induced Pluripotent Stem Cell (iPSC)–Derived Excitatory Pyramidal Neurons (iN) A, Illustration of the rpDCM approach, adapted from Adams et al,^28^ integrating measured excitatory synaptic density iNs as a proxy for synaptic gain to predict EEG power spectra. B, Imaging analysis with neurite segmentation masks based on immunocytochemistry with Synapsin 1 (SYN1, green), β3-tubulin (β3TUB, red), and DAPI nuclei staining (blue). Scale bar = 10 µm. C, SYN1 density in iNs from HC (n = 21) and SCZ (n = 21; total of 107 wells/coverslips from ≥2 independent differentiation batches). D, Predicted EEG power spectra from the applied rpDCM of the translational iPSC cohort (n = 42). E, Distribution of frequency bands in the rpDCM-predicted EEG power spectrum in HC (n = 10) and SCZ (n = 20). F, Intraindividual correlation between rpDCM-predicted gamma1 activity and empirical EEG gamma1 activity of the scalp electrodes C3 (*r* = 0.45; *P* = .048; n = 27), C4 (*r* = 0.43; *P* = .05; n = 27), and F8 (*r* = 0.63; *P* < .001; n = 26). Gray areas = confidence intervals. Illustration of the highlighted electrodes. ^a^SCZ-HC group differences (*P* = .02; 2-tailed linear mixed model). ^b^SCZ-HC group differences (*P* < .01; 2-tailed linear mixed model).

However, despite the consistent link of neuroimaging-based regional structural brain changes with a reduction of synaptic density,^1,10,11^ there remains a mechanistic gap between changes in gene expression levels, potential cellular consequences, and corresponding differences in patient-level intermediate and clinical phenotypes. We hypothesized that variability in synaptic density of patient-derived in vitro neuronal networks constitute a suitable cellular endophenotype linked to individual differences in circuit-level electrophysiology. To test this hypothesis, we modified the DCM approach by Adams and colleagues^28^ who studied different synaptic density parameters in silico to reproduce patient-level EEG power spectrum in a cohort that was independent from our study.^28^ In this rpDCM, we used iPSC-derived neuronal networks to provide empirical assessments of individual synaptic density in vitro to predict the individual rsEEG-PSD levels in vivo (Figure 5A). We analyzed the frequency of the presynaptic bouton marker Synapsin 1 (SYN1) (Figure 5B; eFigure 8A in Supplement 1) of electrophysiologically active iN-derived neuronal networks^18^ as an approximate measure for the density of functional synapses. To this end, SYN1 density within iN neurites was quantified by immunohistochemistry across 42 individuals (21 SCZ, 21 HC). This analysis revealed no differences in cell culture conditions (eFigure 8B and C in Supplement 1) but a significant mean reduction of excitatory synapses by 12.4% in SCZ that was independent of clinical (co)variables of their donors (Figure 5C; eTables 27 and 28 in Supplement 2). We then used the iN-synapse density to simulate the individual-level rsEEG-PSD of the same individual, setting all excitatory synaptic weights in the rpDCM to the empirically observed relative density (Figure 5D; eFigure 8D in Supplement 1). To test model specificity, we varied rpDCM parameters and found that only modulation of all or excitatory synapses were consistent with the EEG findings, but not inhibitory-only or subsets of excitatory synaptic connections (eFigure 9 in Supplement 1).

This rpDCM-simulated rsEEG power spectrum qualitatively recapitulated the groupwise differences of the empirical rsEEG, including the significant differences in the theta and gamma frequency range (Figure 5E; eFigure 10 in Supplement 1). Beyond groupwise differences, we detected nominally (*P* ≤ .05) significant correlations between the individual level of simulated and empirical rsEEG-PSD in the gamma1 range at the C3, C4, and F8 electrodes (Figure 5F; eTables 29 and 30 in Supplement 2), of which the latter passed multiple testing correction (*q* value = 0.017), providing an intrapersonal biophysical link between variability in synaptic density and changes in the rDLPFC’s gamma1 rsEEG-PSD.

## Discussion

This study bridges the translational gap in SCZ by linking synaptic deficits of excitatory neurons to individual changes in brain structure, electrophysiology, and cognitive impairments. Our schizophrenia neurocognition signature identified a pattern of GMV reduction, likely reflecting loss of synapses and dendritic complexity,^1,10,11^ with a pronounced contribution of the rDLPFC. This brain pattern is associated with cognitive impairments and linked to electrophysiological alterations, particularly in the gamma frequency range.

While iPSC modeling is powerful, it remains a time- and cost-intensive bottleneck.^50^ To scale multimodal integration in our deep phenotyped cohorts, we imputed gene expression profiles in silico using genotype data.^27^ We used excitatory iN, which showed substantial gene expression alterations and reduced synapse density in SCZ, to enable the intraindividual translation of genetically driven individual cell–autonomous variability into the individual degree of brain circuitry, electrophysiology, and cognitive changes.

By the reverse adaption of the DCM from Adams and colleagues,^28^ established and trained independently from our cohorts, our rpDCM approach showed that synaptic deficits of excitatory neurons were associated with altered neurophysiology in SCZ. The finding that gamma1-PSD at the F8 electrode—reflecting rDLPFC activity—showed the strongest association with synapse density aligns with the converging evidence across our multimodal study: rDLPFC was prominently involved in the SPLS-derived schizophrenia neurocognition signature and drove EEG–brain score associations. This aligns with the established roles of the rDLPFC^49^ and lower gamma band power^48^ in cognitive impairments in SCZ.

Importantly, our results are in line with the hypothesis that a reduction in excitatory synaptic function progressively contributes to localized cortical disinhibition.^1^ Comparing alternative configurations of our rpDCM revealed that only the modulation of all synapses or the excitatory synapses alone reproduced the rsEEG-PSD group differences, while an inhibitory-only modulation failed to do so, supporting the contribution of excitatory neurons to the observed changes in EEG power. Thus, by linking patient-specific molecular and synaptic deficits of excitatory neurons to intermediate phenotypes of SCZ, our multilayer modeling supports both the long-standing glutamate hypothesis—postulating disrupted excitatory neurotransmission—and Feinberg’s synaptic hypothesis.^1,51^

### Limitations

Despite encouraging findings, several limitations remain. First, modeling was limited to excitatory neurons. Future refinements should incorporate diverse cell types implicated in SCZ,^52,53,54,55^ including fast-spiking GABAergic interneurons—critical for gamma oscillations^48,56^—and glial cells to enrich translational disease modeling. Moreover, the extension of translational phenotype batteries that can be assessed in vivo and in vitro, such as connectivity measurements (eg, task and resting-state functional MRI and multielectrode arrays) or measurement of neurotransmitter concentrations (eg, magnetic resonance spectroscopy, metabolomics), will be an essential prerequisite to further advance this intraindividual translational approach.

Second, while maturation states of iN from the investigated iPSC donors were comparable, future longitudinal studies will be needed to determine whether the identified molecular and synaptic deficits arise during early development or later maturation.

Third, iPSC models do not account for environmental contributions to SCZ, such as perinatal infections, nutritional deficits, substance use, trauma, and stress,^57^ which may interact with genetic predisposition to shape brain structure and function. Our results suggest that synaptic pathology contributes upstream to these macro-scale changes, but a more complete understanding requires integration of environmental variables.

Fourth, despite the power of our genetic imputation approach, its generalizability remains poor and larger iN cohorts are required to improve the explanatory capacity and generalizability of this strategy. Thus, the establishment of substantially larger translational deep phenotype cohorts with matching iPSCs (N >200) will be instrumental to obtain sufficient detection power and mitigate the high level of clinical heterogeneity in SCZ. Furthermore, the generalizability of our developed models is still limited, which might be due to different phenotyping protocols between the cohorts, different MRI scanners, and experimental variability at the level of iPSC cultivation and differentiation.

## Conclusions

In summary, our findings in this genetic association study support an integrated model in which genetic predisposition, acting through altered expression of synaptic genes, leads to reduced excitatory synaptic density in key cortical and subcortical regions. These cellular-level changes of synapse density, potentially amplified by environmental factors, then contribute to widespread structural and functional brain alterations, including reduced gamma band power, and thereby contribute to cognitive impairment in SCZ.

This proof-of-concept study establishes a generally applicable strategy to bridge the gap between cellular endophenotypes, modeled in vitro, and in vivo clinical intermediate phenotypes. Linking iPSC-derived cellular data with clinical imaging, EEG, and cognitive profiles by the use of machine learning strategies and rpDCM, we outline a strategy for future biomarker discovery and precision psychiatry. Ultimately, multilayered translational frameworks could accelerate development of targeted, mechanism-based treatments for cognitive dysfunction in SCZ.

## Notes

Supplement 1. eFigure 1. SPLS ‘Aging Signature’ and Characterization of the ‘Schizophrenia Neurocognition Signature’ eFigure 2. Association of ‘Schizophrenia Neurocognition Signature’ With Functional Outcome eFigure 3. Association of Polygenic Risk With ‘Schizophrenia Neurocognition Signature’ eFigure 4. Quality Control, Imputed and Empirical Gene Expression in iPSC-Derived Excitatory Neurons in Schizophrenia eFigure 5. Model Quality of hiPSC-Based Neuronal Transcriptome Prediction of Individual Cognition and Brain Scores eFigure 6. Structural rDLPFC Changes Within ‘Schizophrenia Neurocognition Signature’ and Their Relation to Cognitive Performance eFigure 7. EEG Power Spectrum Alterations of Gamma 1 Frequency Band Relate to the Individual Molecular Signature eFigure 8. High-Resolution Imaging to Determine Synaptic Density and Synaptic Gain Modulation in Dynamic Causal Model eFigure 9. Sensitivity Analysis Dynamic Causal Model eFigure 10. Comparing Band Power Alterations Derived From Reverse Personalized Dynamic Causal Model (rpDCM) and Empirical EEG Alterations in Schizophrenia eMethods. Supplemental Methods eReferences.

Supplement 2. eTable 1. Cohort Characteristics eTable 2. SPLS model: results eTable 3. SPLS model: phentypic pattern eTable 4. SPLS model: Anatomical Features of the brain signatures, part 1 eTable 5. SPLS model: Anatomical Features of the brain signatures, part 2 eTable 6. iN DEGs eTable 7. SVRmodel iN DEG - Cognition (omega) and Brain (epsilon) scores: Performance Cohort 1 (Training), Cohort 2(Validation), iPSC cohort (Validation) eTable 8. SVRmodel iN DEG - Cognition (omega) and Brain (epsilon) scores: Predictions eTable 9. SVRmodel: Association of Cognition and Brain Scores with clinical covariates eTable 10. SVRmodel: Performance 100 Random iN Gene Sets eTable 11. SVRmodel: Performance postmortem DLPFC gene expression model eTable 12. iN DEGs: GO BP terms eTable 13. MAGMA analysis eTable 14. EEG analysis: SSD vs. HC eTable 15. SVRmodel: EEG-based prediction of Cognition (omega) and Brain scores (epsilon) -MODEL PERFORMANCE eTable 16. SVRmodel: EEG-based prediction of Cognition (omega) and Brain scores (epsilon) - PREDICTIVE ELECTRODES eTable 17. SVRmodel: EEG-based prediction of Brain scores (epsilon) - all electrodes eTable 18. SVRmodel: EEG-based prediction of Brain scores (epsilon) - F8 electrode eTable 19. SVRmodel: EEG-based prediction of Cognition scores (omega) - all electrodes eTable 20. SVRmodel: EEG-based prediction of Cognition scores (omega) - F8 electrode eTable 21. SVRmodel: iN DEG - EEG - MODEL PERFORMANCE eTable 22. SVRmodel: iN DEG - EEG_Theta all electrodes eTable 23. SVRmodel: iN DEG - EEG_Gamma 1 all electrodes eTable 24. SVRmodel: iN DEG - EEG_Gamma 2 all electrodes eTable 25. SVRmodel: iN DEG - EEG_Gamma 1 F8 eTable 26. SVRmodel: iN DEG - EEG_Clinical covariates eTable 27. Synapse Density per donor eTable 28. Synapse Density: association with clincial covariates eTable 29. rpDCM: input and predicted EEG results eTable 30. rpDCM: correlation predicted EEG with empirical EEG pattern_per electrode

Supplement 3. CDP Working Group

Supplement 4. Data Sharing Statement
