# Supplementary material for: Bridging the Scales via Personalized Cellular Modeling and Deep Phenotyping in Schizophrenia
Source: JAMA Psychiatry. 2026 Mar 28;83(5):510–22. doi: 10.1001/jamapsychiatry.2026.0576 (PMC13033174; doi:10.1001/jamapsychiatry.2026.0576)
Supplement: Supplement 1. — eFigure 1. SPLS ‘Aging Signature’ and Characterization of the ‘Schizophrenia Neurocognition Signature’ eFigure 2. Association of ‘Schizophrenia Neurocognition Signature’ With Functional Outcome eFigure 3. Association of Polygenic Risk With ‘Schizophrenia Neurocognition Signature’ eFigure 4. Quality Control, Imputed and Empirical Gene Expression in iPSC-Derived Excitatory Neurons in Schizophrenia eFigure 5. Model Quality of hiPSC-Based Neuronal Transcriptome Prediction of Individual Cognition and Brain Scores eFigure 6. Structural rDLPFC Changes Within ‘Schizophrenia Neurocognition Signature’ and Their Relation to Cognitive Performance eFigure 7. EEG Power Spectrum Alterations of Gamma 1 Frequency Band Relate to the Individual Molecular Signature eFigure 8. High-Resolution Imaging to Determine Synaptic Density and Synaptic Gain Modulation in Dynamic Causal Model eFigure 9. Sensitivity Analysis Dynamic Causal Model eFigure 10. Comparing Band Power Alterations Derived From Reverse Personalized Dynamic Causal Model (rpDCM) and Empirical EEG Alterations in Schizophrenia eMethods. Supplemental Methods eReferences. [file jamapsychiatry-e260576-s001.pdf]

Supplemental Online Content

Raabe FJ, Popovic D, Vetter C, et al; CDP Working Group. Bridging the scales via personalized cellular modeling and deep phenotyping in schizophrenia. *JAMA Psychiatry*. Published online March 28, 2026. doi:10.1001/jamapsychiatry.2026.0576

eFigure 1. SPLS 'Aging Signature' and Characterization of the 'Schizophrenia Neurocognition Signature'

eFigure 2. Association of 'Schizophrenia Neurocognition Signature' With Functional Outcome

eFigure 3. Association of Polygenic Risk With 'Schizophrenia Neurocognition Signature'

eFigure 4. Quality Control, Imputed and Empirical Gene Expression in iPSC-Derived Excitatory Neurons in Schizophrenia

eFigure 5. Model Quality of hiPSC-Based Neuronal Transcriptome Prediction of Individual Cognition and Brain Scores

eFigure 6. Structural rDLPFC Changes Within 'Schizophrenia Neurocognition Signature' and Their Relation to Cognitive Performance

eFigure 7. EEG Power Spectrum Alterations of Gamma 1 Frequency Band Relate to the Individual Molecular Signature

eFigure 8. High-Resolution Imaging to Determine Synaptic Density and Synaptic Gain Modulation in Dynamic Causal Model

eFigure 9. Sensitivity Analysis Dynamic Causal Model

eFigure 10. Comparing Band Power Alterations Derived From Reverse Personalized Dynamic Causal Model (rpDCM) and Empirical EEG Alterations in Schizophrenia

eMethods. Supplemental Methods

eReferences.

© 2026 Raabe FJ et al. *JAMA Psychiatry*.

This supplemental material has been provided by the authors to give readers additional information about their work.

© 2026 Raabe FJ et al. *JAMA Psychiatry*.

eFigure 1. SPLS 'Aging Signature' and Characterization of the 'Schizophrenia Neurocognition Signature'

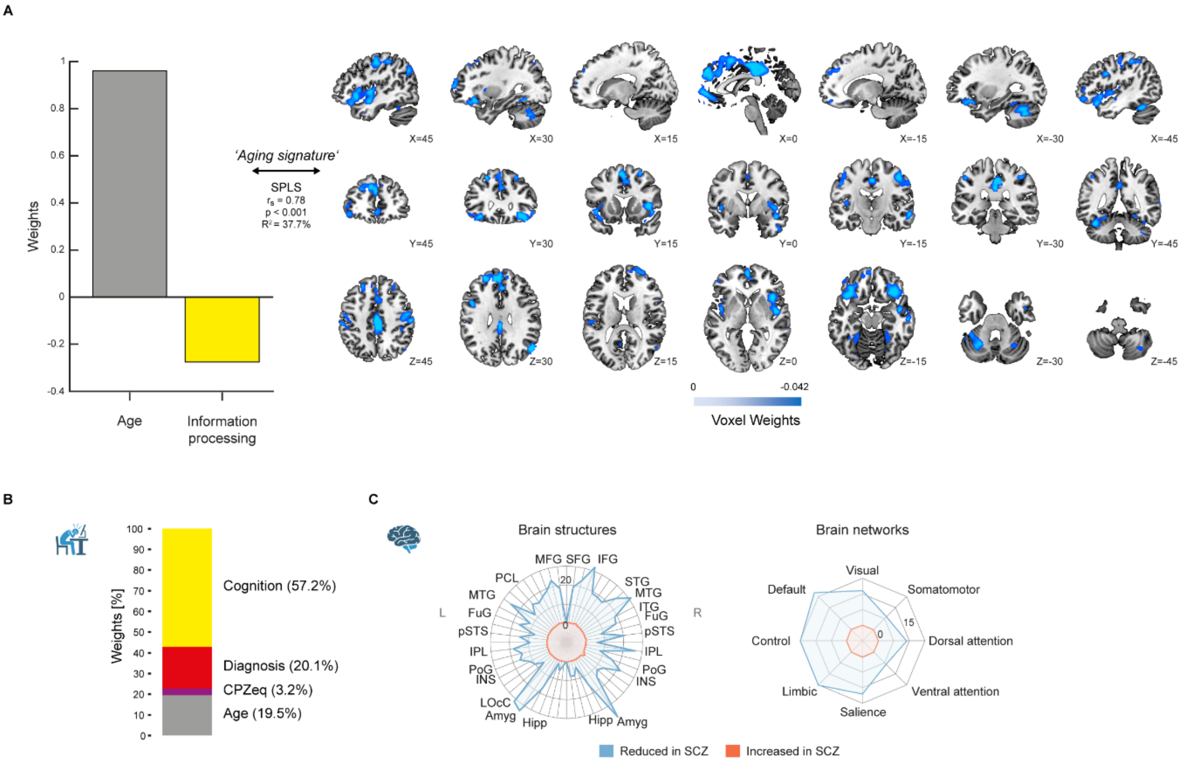

A, Left: The barplot visualizes the phenotypic pattern of the '*aging signature*' contained in Latent Variable 1 (LV1), derived by the multivariate sparse partial least squares (SPLS) algorithm on cohort 1 (N = 131, r = 0.78, $p < 0.001$, R^2 = 37.7%). Right: Illustration of the brain pattern of the '*aging signature*' contained in Latent Variable 1 (LV1), derived by SPLS algorithm (N = 131). Negative weighting in the blue color scale. The brain patterns were visualized in the MNI152 standard space using the open-source 3D rendering software Connectome Workbench v1.4.2. (https://humanconnectome.org/software/connectome-workbench).

B, Bar plot illustrating the accumulated significant contribution of cognition (yellow), diagnoses (red), CPZ equivalent (violet), and age (grey) to the 'schizophrenia neurocognition signature'.

C, Spider plots highlight mapping onto *(upper plot)* the top neuroanatomic regions[1,2] and *(plot below)* 8 large-scale functional brain networks[3,4] of the brain pattern captured by the *'schizophrenia neurocognition signature'*, depicting the proportion of positively (red) and negatively (blue) weighted voxels in each region and networks. Arrow indicates location of the right dorsolateral prefrontal cortex (rDLPFC). *Abbreviations: Amyg, Amygdala; FuG, Fusiform Gyrus; Hipp, Hippocampus; IFG, Inferior Frontal Gyrus; INS, Insular Gyrus; IPL, Inferior Parietal Lobule; ITG, Inferior Temporal Gyrus; LOcC, Lateral Occipital Cortex; MFG, Middle Frontal Gyrus; MTG, Middle Temporal Gyrus; PoG, Postcentral Gyrus; pSTS, Posterior Superior Temporal Sulcus; STG, Superior Temporal Gyrus.*

© 2026 Raabe FJ et al. *JAMA Psychiatry*.

eFigure 2. Association of 'Schizophrenia Neurocognition Signature' With Functional Outcome

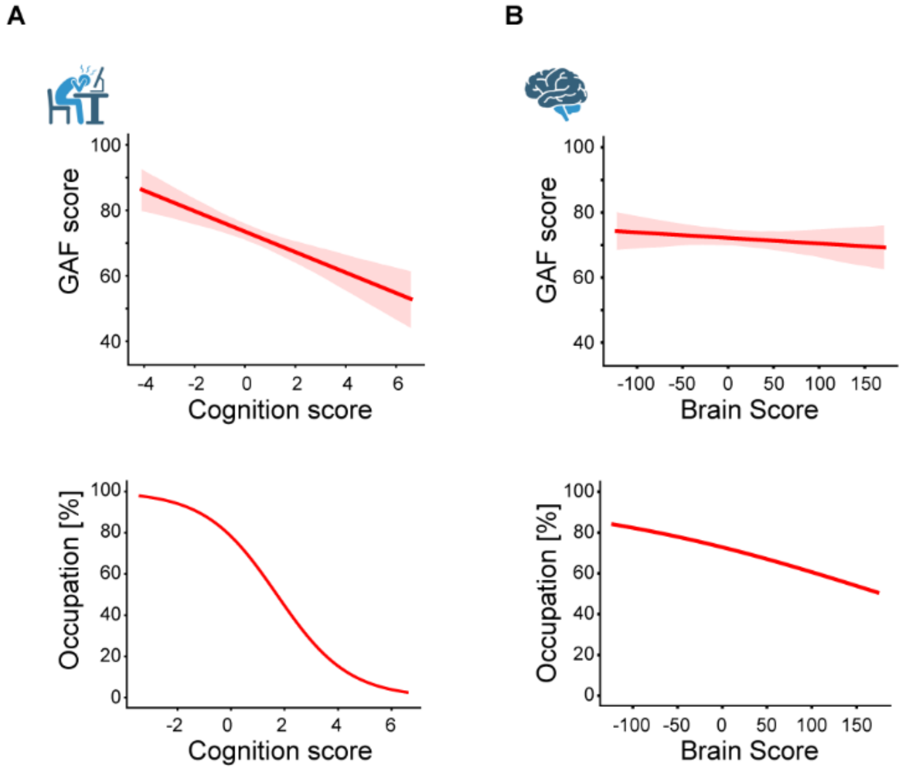

A, Upper panel: Pearson's correlation analysis of Global Assessment of Functioning (GAF) Score (y-axis) and *Cognition score* (x-axis) of the '*schizophrenia neurocognition signature'* across both cohorts ($r = -0.292$, $p < 0.001$) with respective regression line. Lower panel: Logistic regression analysis of the association between *Cognition score* (x-axis) and the occupational status (y-axis; coefficient = -0.748, $p < 0.001$, pseudo $R^2 = 0.206$)

B, Upper panel: Pearson's correlation analysis of Global Assessment of Functioning (GAF) Score (y-axis) and *Brain score* of the '*schizophrenia neurocognition signature'* (x-axis) across both cohorts ($r = -0.06$, $p = 0.38$; not significant) with respective regression line. Lower panel: Logistic regression analysis of the association between *Brain score* (x-axis) and the occupational status (y-axis; coefficient = -0.00554, $p = 0.019$, pseudo $R^2 = 0.023$).

eFigure 3. Association of Polygenic Risk With 'Schizophrenia Neurocognition Signature'

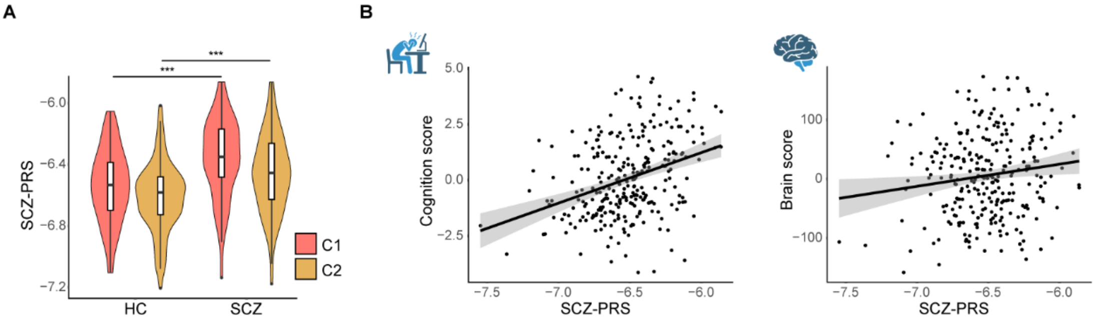

A, Group-level comparison of the SCZ polygenic risk score (SCZ-PRS) based on the PGC wave 3[5] between HC (N_{C1}=65, N_{C2}=84) and SCZ (N_{C1}=52 N_{C2}=76) individuals in the C1 (red) and C2 (orange) using the Wilcoxon-sum rank test (*** = $p < 0.001$).

B, Correlation analysis of SCZ-PRS (x-axis) and *Cognition* (left, y-axis) or *Brain score* (right, y-axis) across both cohorts (N=272). Black lines indicate regression line based on linear models correcting for the first three ancestry principal components for the *Cognition score* (r=0.36, p-value=2.34e-09, t-test) and the *Brain score* (r=0.16, p-value=0.0131). Grey shading indicates estimated standard error.

© 2026 Raabe FJ et al. *JAMA Psychiatry*.

eFigure 4. Quality Control, Imputed and Empirical Gene Expression in iPSC-Derived Excitatory Neurons in Schizophrenia

© 2026 Raabe FJ et al. *JAMA Psychiatry*.

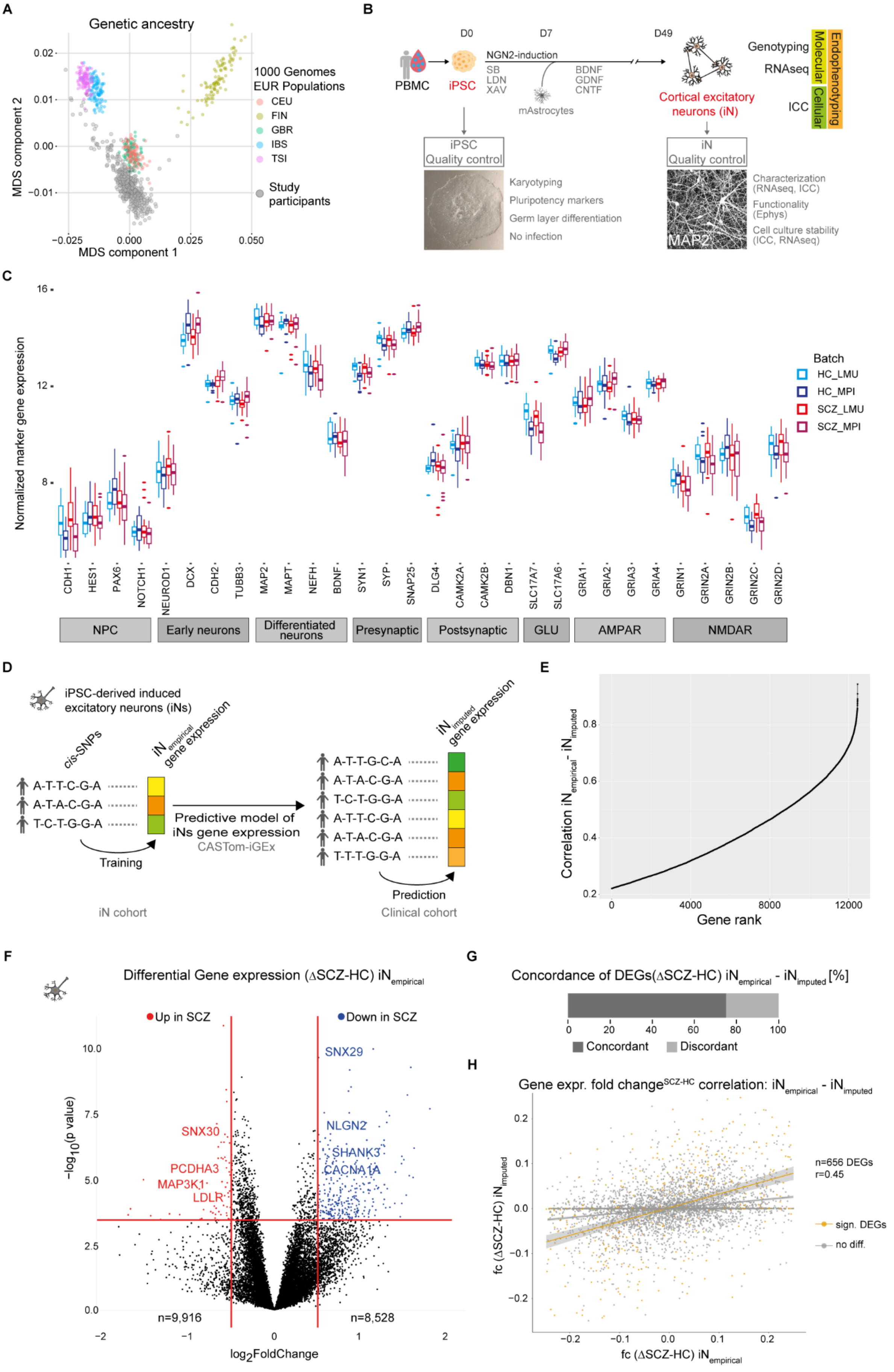

© 2026 Raabe FJ et al. *JAMA Psychiatry*.

A, Plot of Multidimensional scaling (MDS) components analysis of the genotyped study cohorts combined with 1000 Genomes project populations.

B, Schematic overview of the experimental iPSC-approach of directed neuronal differentiation. Extended quality control analysis are reported in the supplemental data of the companion study Raabe et al.[6]

C, Expression level of neuronal subtype and maturation stage specific genes based on iN RNA-Seq at day 49 across HC (n=36 independent iPSC lines) and SCZ (n=52 independent iPSC lines) and across 4 batches and 2 different experimental sites (LMU, MPI, n=88 independent iPSC lines) of iN differentiation. Marker genes for neural precursor genes (NPC): CDH1, HES1, PAX6, NOTCH1; early neurons: NEUROD1, DCX, CDH2, TUBB3; differentiated neurons: MAP2, MAPT, NEFH, BDNF; Pre-synaptic marker genes: SYN1, SYP, SNAP25; Post-synaptic marker genes: DLG4, CAMK2A, CAMK2B, DBN1; Glutamatergic (GLU) marker genes: SLC17A7, SLC17A6; AMPAR marker genes: GRIA1, GRIA2, GRIA3, GRIA4; NMDAR marker genes: GRIN1, GRIN2A, GRIN2B, GRIN2C, GRIN2D.

D, Scheme illustrates predictive model to impute gene expression from genetic variants in iPSC-derived induced excitatory neurons (iNs). The model is trained using a dataset from an iPSC cohort[6] and subsequently applied to predict gene expression in the clinical cohort based on their genotypes.

E, Rank curve illustrates the distribution of correlation coefficients between imputed and empirically measured gene expression across the transcriptome of iNs from n=79 donors.

F, Volcano plot showing the log2 fold-change (x-axis) and significance (-log10 p-value, y-axis) of differentially expressed genes (DEGs) between iNs derived from SCZ (N=35) and HC (N=29) iPSC lines at day 49. Positive fold-changes indicate lower expression in SCZ. Red/blue dots indicate significance (p(FDR)<=0.01) and minimal fold-change (|fc|>=0.4) cutoffs to define DEGs. Text highlights selected DEGs.

G, Bar plot illustrates concordant overlap (dark grey) of imputed with empirical iN DEGs (ΔSCZ-HC).

H, Correlation of gene expression fold changes (fc) between SCZ and HC in empirical and imputed gene expression. Line in orange represents the Pearson correlation coefficient r (r=0.4468781, p-value: < 2.2e-16, n=656) for the fold change of significant differentially expressed genes (DEGs), while the dark grey line indicates the correlation coefficient for non-significant DEGs (r=0.2501, p-value: < 2.2e-16, n=4870). Each line is accompanied by a confidence interval, shaded in light grey.

© 2026 Raabe FJ et al. *JAMA Psychiatry*.

eFigure 5. Model Quality of hiPSC-Based Neuronal Transcriptome Prediction of Individual Cognition and Brain Scores

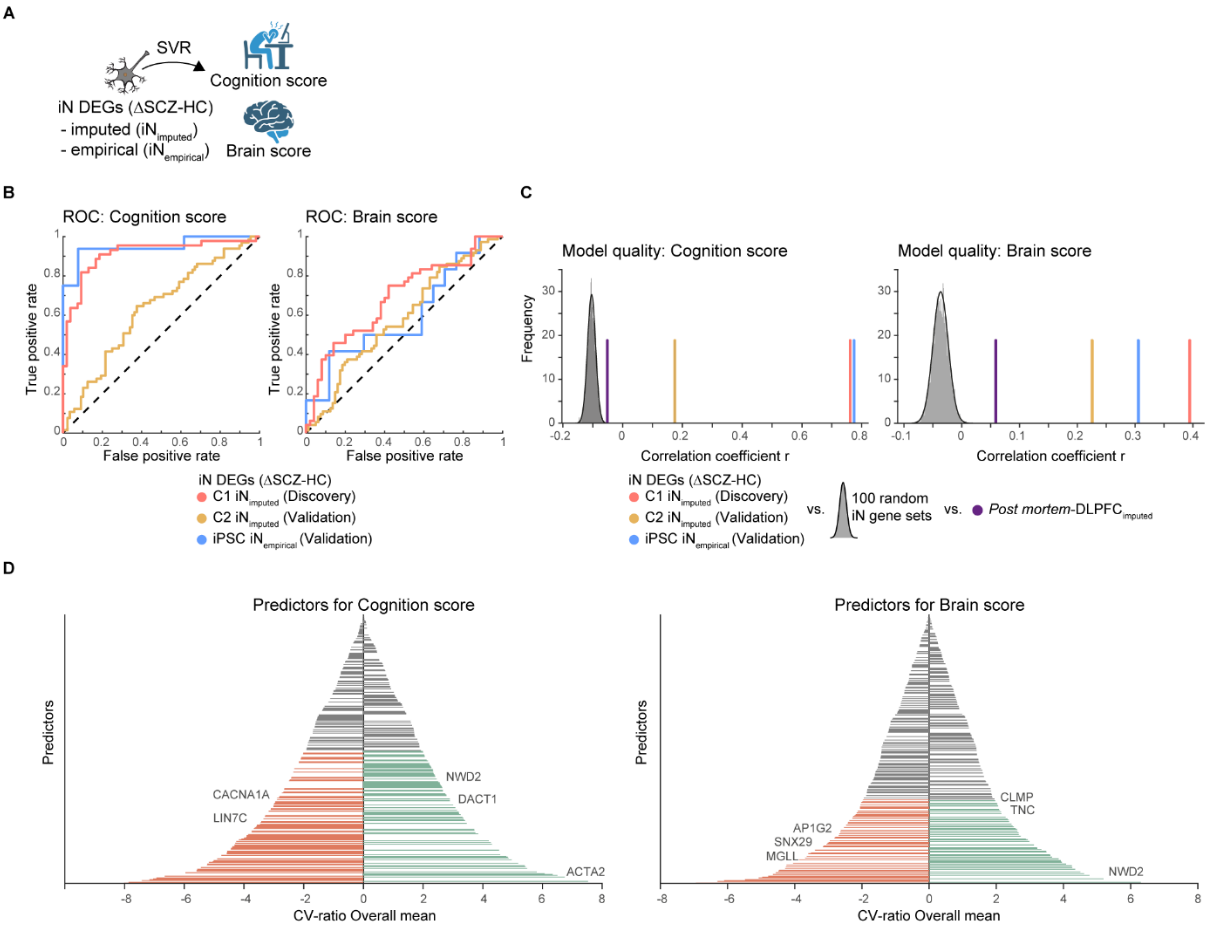

A, Illustration of the nested cross-validated support vector regression (SVR) prediction of the individual *Cognition* and *Brain scores* of the *'schizophrenia neurocognition signature'* based on imputed gene expression levels of 310 DEGs (ΔSCZ-HC) in iPSC-derived iNs ($iN_{imputed;}$ see **eFigure 4**) in the discovery cohort 1 (C1 $iN_{imputed}$, red) und separate imputed validation cohort 2 (C2 $iN_{imputed}$, orange) and held-out empirically measured (not-imputed) iN transcriptome data of the iPSC cohort (iPSC $iN_{empirical}$, blue).

B, Receiver operating characteristic (ROC) curves and mean area under the curve (AUC) values describing the SVR performance for all models (**Figure 2A**) to predict the *Cognition* (left) and the *Brain score* (right) in the discovery (C1, red, $AUC_{Cognition\ score}$=0.91, $AUC_{Brain\ score}$=0.69), validation (C2, orange, $AUC_{Cognition\ score}$=0.63, $AUC_{Brain\ score}$=0.59) and empirical validation (iN, blue, $AUC_{Cognition\ score}$=0.95, $AUC_{Brain\ score}$=0.60) cohort.

C, Permutation based evaluation of feature specificity of the SVR model, showing the frequency (y-axis) of the Spearman correlation coefficient (r, x-axis) between the observed and predicted *Cognition* (left) or *Brain scores* (right) using the same SVR strategy but based on 100 randomly generated iN gene feature sets (grey bars) as empirical background distribution and DLPFC (post mortem) gene expression levels (violet bar) using our previously published model.[27] SVR model results from **Figure 2A** are shown as bars for comparison across the three cohorts (C1: red, C2: orange, iN: blue).

D, Feature importance plots of the SVR models depicting the ranked cross-validation ratio overall means (CVR-OM, y-axis) of all 310 DEGs gene features (y-axis) in the SVR model to predict the

© 2026 Raabe FJ et al. *JAMA Psychiatry*.

Cognition (left) or *Brain scores* (right). Significant features are colored with annotations for selected genes of interest.

© 2026 Raabe FJ et al. *JAMA Psychiatry*.

eFigure 6. Structural rDLPFC Changes Within 'Schizophrenia Neurocognition Signature' and Their Relation to Cognitive Performance

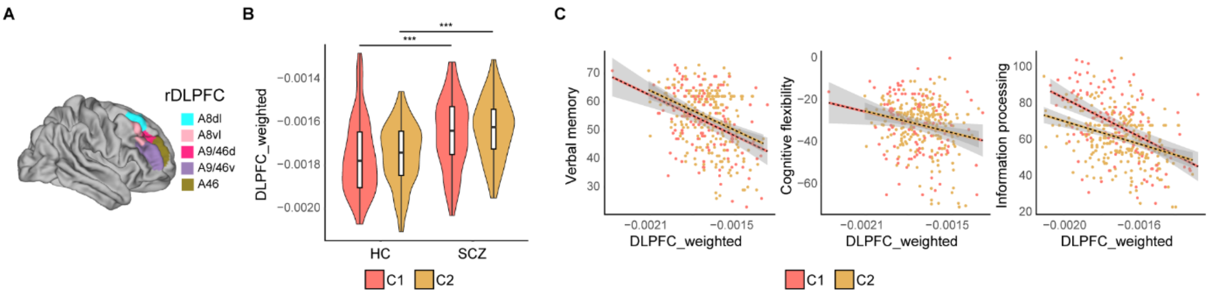

A, Illustration anatomical regions included in the rDLPFC analysis.

B, Distribution of GMV averaged across all voxels within the right dorsolateral prefrontal cortex (rDLPFC; illustration highlights the considered anatomical regions) weighted by the voxel level feature weight in the SPLS for each individual separated by cohort for individuals with SCZ and HC. *** indicates significant group comparison in C1 (red; p-value=0.00024) and C2 (orange; p-value= 1.497e-09) using the Wilcoxon-sum rank test.

C, Correlation of weighted GMV in the right DLPFC (x-axis) and performance in different cognitive domains (y-axis, Verbal memory, Cognitive flexibility, Information processing) in C1 and C2 (Verbal memory: r_{C1}=-0.35, p-value$_{C1}$=5.34e-05, r_{C2}=-0.37, p-value$_{C2}$=7.33e-10; Cognitive flexibility: r_{C1}=-0.30, p-value$_{C1}$=0.000645, r_{C2}=-0.21, p-value$_{C2}$=0.000654; Information processing: r_{C1}=-0.30, p-value$_{C1}$=0.000645, r_{C2}=-0.368, p-value$_{Cohort2}$=1.04e-09).

© 2026 Raabe FJ et al. *JAMA Psychiatry*.

eFigure 7. EEG Power Spectrum Alterations of Gamma 1 Frequency Band Relate to the Individual Molecular Signature

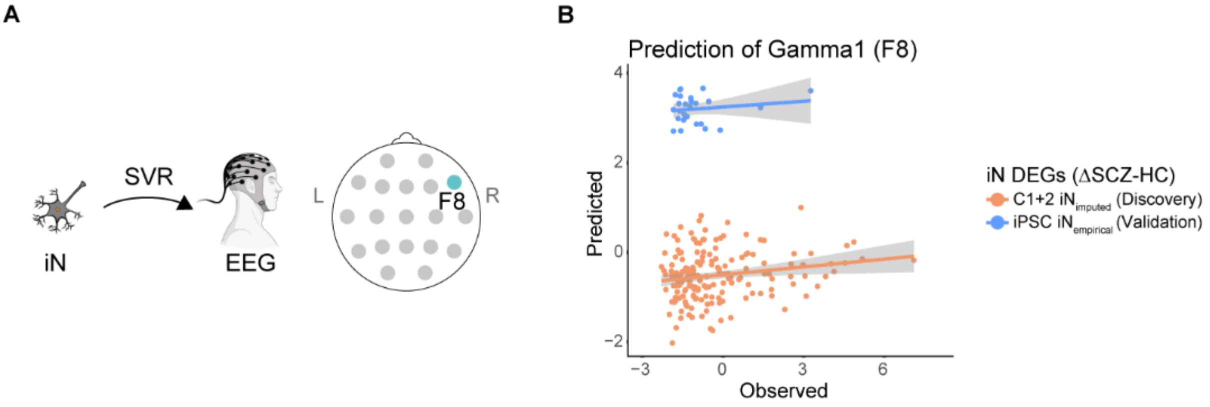

A, Illustration of the prediction of the individual EEG frequency power spectra of Gamma 1 of F8 electrode using SVRs from genotype imputed gene expression profiles in iPSC-derived iN of independently selected genes as features.

B, Correlation analysis between the individual level predicted (y-axis) and observed (x-axis) absolute EEG frequency power average over the F8 electrode in gamma 1 band power. Prediction of the band power was performed using support vector regression (SVR) from genotype imputed gene expression levels of 310 iN DEGs (ΔSCZ-HC) in a fused subset of individuals from cohort C1 and C2 with available EEG Data (C1+2 iN $DEG_{imputed}$; N=178, discovery, orange). Replication sample from a subset of held out individuals of the iPSC cohort with empirically measured iN DEGs (iPSC iN $DEG_{empirical}$; N=26; validation, blue). The SVR model was trained using 5-fold cross validation and all predicted values are based on the held-out sample subsets. Lines indicate linear regression model for discovery cohort and replication sample predicting the gamma 1 band power (r_D=0.19., p_D(permutation-test)=0.006, r_{iN}=0.15, p_{iN}(permutation-test)=0.23, p_{iN}(mean average error)=0.0008), showing the iPSC replication sample with a different offset for better visibility.

© 2026 Raabe FJ et al. *JAMA Psychiatry*.

eFigure 8. High-Resolution Imaging to Determine Synaptic Density and Synaptic Gain Modulation in Dynamic Causal Model

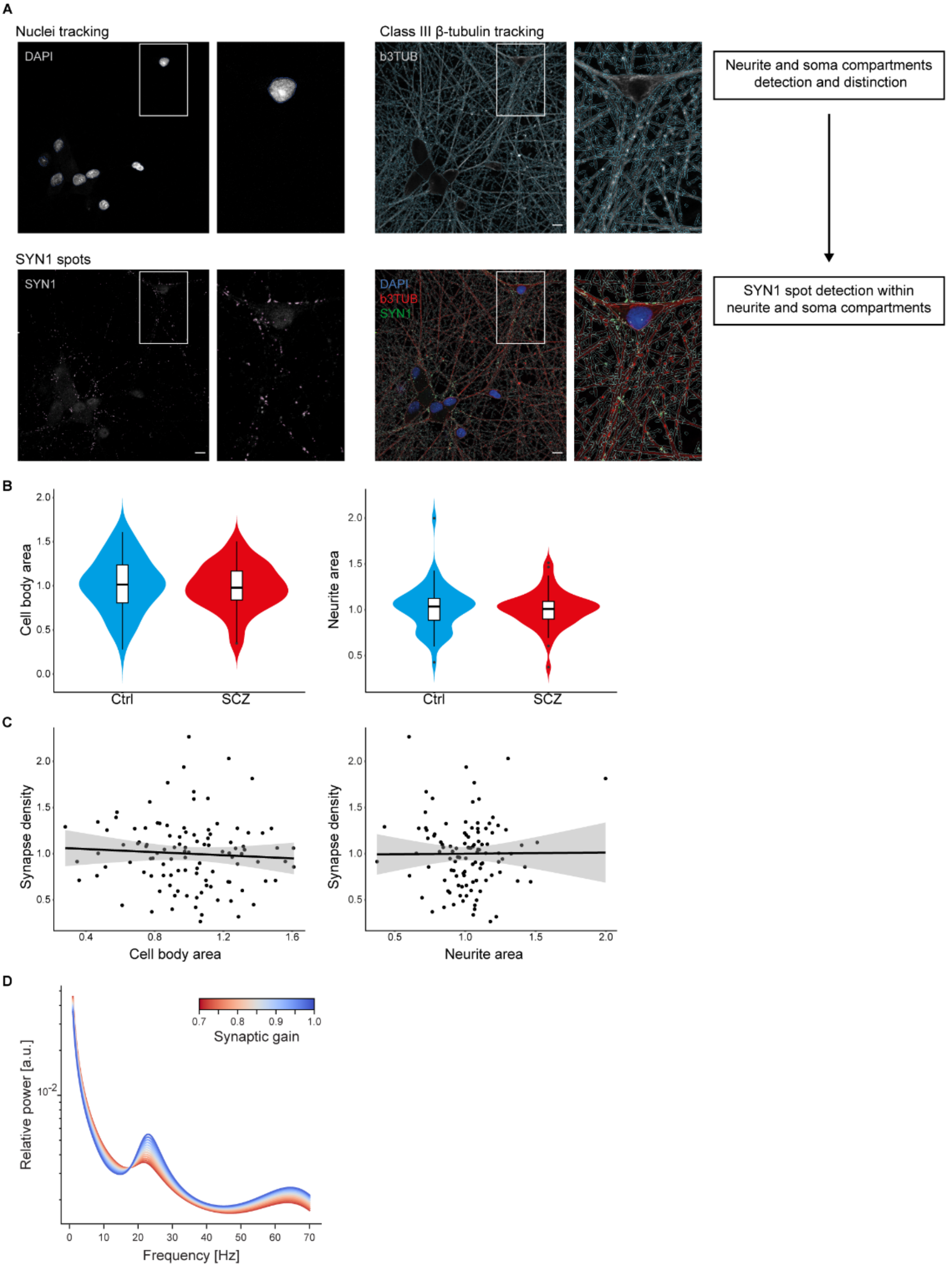

© 2026 Raabe FJ et al. *JAMA Psychiatry*.

A, Representative examples of the applied high-resolution confocal-microscopy images analysis pipeline with the original florescence channels and the respective mask. White rectangles indicate position of the respective high magnification field of view. Scale bar indicates 10 µm.

B, Violin plot illustrates the (left) total cell body area and the (right) total neurite area based on immunocytochemistry of neurites (b-Tubulin) and cell nuclei (DAPI) based soma detection using high content imaging and confocal microscopy across iN cell culture from 42 individuals across 107 different wells from 5 differentiation batches (N=21 HC, N=21 SCZ). Statistical significance of differences between HC and SCZ from a two-tailed linear mixed model comparison is indicated by an * ($p\text{-value}_{\text{neurite area}} = 0.858$; $p\text{-value}_{\text{cell body area}} = 0.826$).

C, Correlation of synapse density and (left) total cell body as well as (right) total neurite area based on immunocytochemistry of synapses (Synapsin), neurites (b-Tubulin) and cell nuclei (DAPI) using high content imaging and confocal microscopy across iN cell culture from 42 individuals (N=21 HC, N=21 SCZ). Statistical significance of the correlation evaluated with Pearson (Synapse – cell body: r= −0.064, p-value = 0.41; Synapse – neurite: r = 0.0075, p = 0.651).

D, Graph illustrates the application of the Dynamic Causal Model (DCM) by Adams et al.[7] to predict the EEG power spectrum with synaptic gain changes varied in increments of 0.05. The plot shows the effect of changing synaptic gain on the relative power across the EEG power spectrum, indicated by the blue and red lines which represent different levels of gain adjustment.

© 2026 Raabe FJ et al. *JAMA Psychiatry*.

eFigure 9. Sensitivity Analysis Dynamic Causal Model

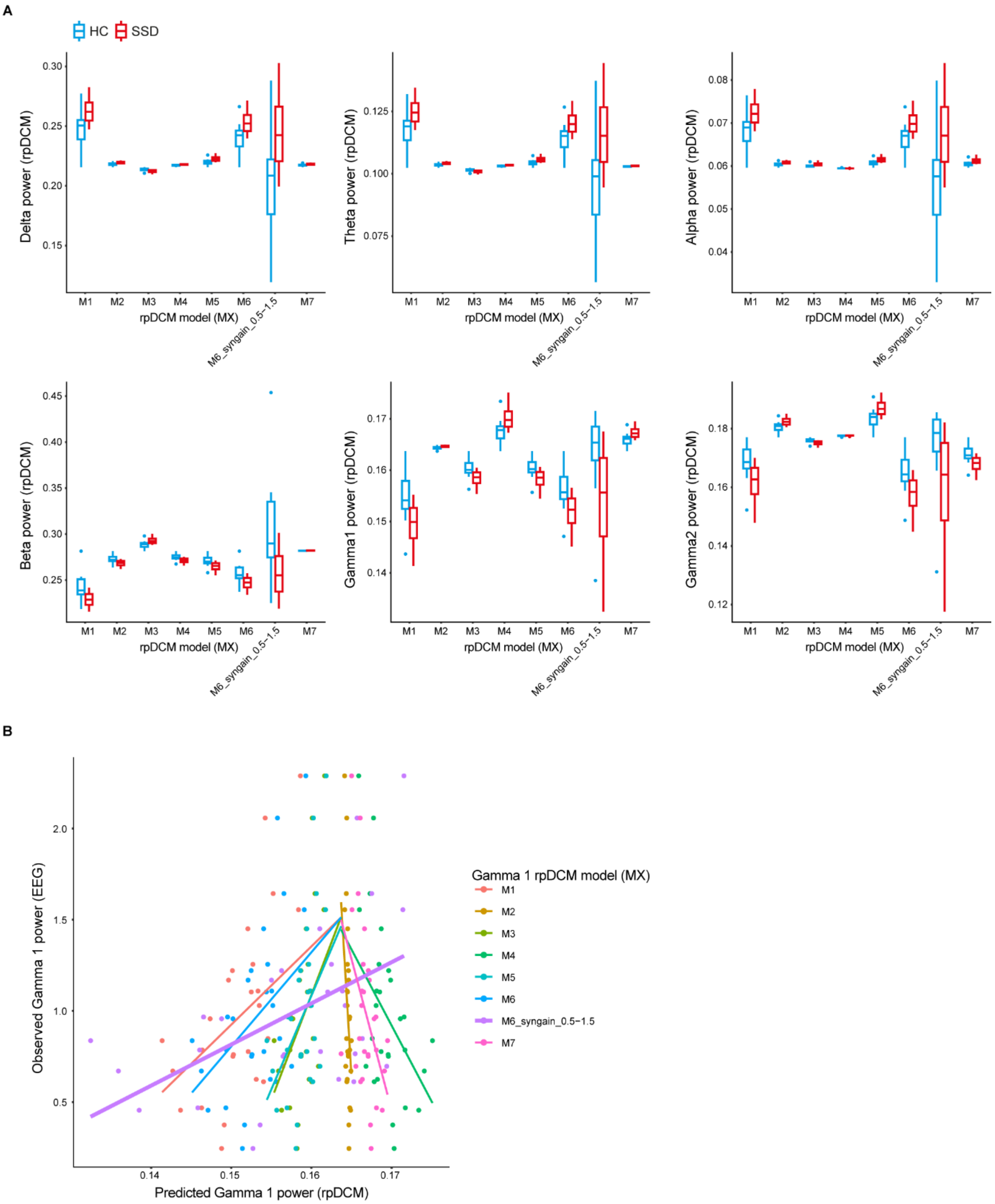

A, Boxplot of power spectrum (y-axis) distribution predicted by different versions of the reverse personalized Dynamic Causal Model (rpDCM; x-axis) based on iN-derived synaptic density measurements across healthy controls (HC, N=10) and SSD individuals (N=18). Evaluated models include original 5 models tested by Adams et al. 2022,[7] model 1 (M1) decreasing all synaptic gain parameters in the DCM, model 2 (M2) reducing only excitatory input to inhibitory neurons, model 3 (M3) reducing only self-inhibition of inhibitory neurons, model 4 (M4) increasing self-inhibition of inhibitory neurons, model 5 (M5) increasing only self-excitation on pyramidal neurons, model 6

© 2026 Raabe FJ et al. *JAMA Psychiatry*.

(M6) - used in the main analysis, see **Figure 5** - modulating all excitatory connections according to the iN measurements mapping the synaptic density to the synaptic gain interval 0.7-1, M6 mapping the synaptic density measurements to the synaptic gain interval 0.5-1.5 (M6_syngain_0.5-1.5), and a model modulating all inhibitory synaptic connections in the DCM according to iN synapse density parameters (M7).

B, Regression analysis of measured gamma1 power (y-axis) on predicted gamma1 power (x-axis) at the F8 electrode based on all models 1-7 from A.

© 2026 Raabe FJ et al. *JAMA Psychiatry*.

eFigure 10. Comparing Band Power Alterations Derived From Reverse Personalized Dynamic Causal Model (rpDCM) and Empirical EEG Alterations in Schizophrenia

A

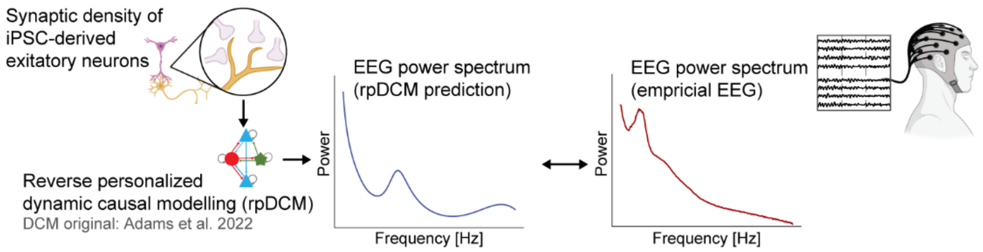

B

	θ	γ1	γ2
sign. ΔrsEEG (SCZ-HC) frequency bands (rpDCM prediction)	↑	↓	↓
sign. ΔrsEEG (SCZ-HC) frequency bands (empirical EEG)	↑	↓	↓

A, Illustration rpDCM approach, adapted from Adams et al. (2022)[7], integrating measured excitatory synaptic density from iPSC-derived induced excitatory neurons (iNs) of patients with schizophrenia (SCZ) and healthy controls (HC) as a proxy for synaptic gain to generate predictive EEG power spectra for individuals of the iPSC cohort and their comparison with empirical EEG-recordings within iPSC cohort.

B, Table highlights the directional changes of in frequency band power between SCZ and HC based on the rpDCM prediction (top) and the empirical EEG recoding (below). Frequency bands for which original DCM28 510 was not optimized are visualized with a transparency level of 50%.

© 2026 Raabe FJ et al. *JAMA Psychiatry*.

eMethods. Supplementary Methods

Participant Recruitment and definition of analysis groups

Participants of Cohort 1 (C1) were part of the *'Multimodal Imaging in Chronic Schizophrenia Study'* (*MIMICSS*) was a pilot study based on the longitudinal *PsyCourse* study,[8] and was performed at the Department of Psychiatry, University Hospital, LMU Munich, Germany (see supplements from[9]). This study was approved by the local ethics committee of Faculty of Medicine, LMU Munich (project number: 17–13). The patients who provided written informed consent were diagnosed by two independent, experienced psychiatrists using the criteria of the International Statistical Classification of Diseases and Related Health Problems, 10th revision (ICD-10). Beside patients also unaffected healthy controls (HCs) – with a subgroup of first-degree unaffected relatives (URs) – with no current or past mental illness according to the Mini International Neuropsychiatric Interview (M.I.N.I.)[10] were recruited.

Cohort 2 (C2) originates from the *'Clinical Deep Phenotyping study'* (*CDP*), study protocol was described in details previously,[9] which is a multimodal add-on study of the Munich Mental Health Biobank (ethics project number 18-716).[11] The study was registered at the German Clinical Trials Register (DRKS, ID: DRKS00024177) and received approval from the local ethics committee of the Faculty of Medicine, LMU Munich (project numbers 20-0528 and 22-0035). It encompasses patients as well as HCs – with a subgroup of first-degree unaffected relatives (URs) – who had no lifetime psychiatric disorders, all classified by the M.I.N.I.[10] according to the DSM-5, text revision (DSM-5-TR, Version 7.0.2) after providing written informed consent.

For this study, we included only patients with schizophrenia spectrum disorders (SSD), mainly with a diagnosis of schizophrenia (SCZ), and healthy controls (HCs) with a subgroup of unaffected relatives (URs) from C1 and C2. Psychotic symptom severity was determined with the Positive and Negative Syndrome Scale (PANSS).[12] Cognitive performance was determined by trained study staffs with several task covering diverse domains of cognitive performance as follows:

Participants of C1 (*MIMICSS*) completed a neuropsychological test battery covering the following cognitive domains: *Episodic verbal memory*, measured using the Verbal Learning and Memory Test (VLMT);[13,14] *Working memory*, assessed with the Digit Span Test (DST, a subtest of the *Hamburg-Wechsler Intelligence Test*);[15] *Information processing*, evaluated using Digit Symbol Substitution Test (DSST, a subtest of the *Hamburg-Wechsler Intelligence Test*);[15] *Cognitive flexibility*, assessed with the Trail Making Test (TMT),[16] specifically the time difference between Part B and Part A; *Crystalline intelligence*, assessed using German vocabulary test (German: *Wortschatztest* (WST),[17] with the normalized number of correctly recognized words converted into a verbal IQ score.

For C2 (*CDP*), cognitive performance was assessed using the *Brief Assessment of Cognition in Schizophrenia* (BACS)[18] and the TMT. Specifically, *verbal memory* was measured using the BACS Verbal Memory subtest (equivalent to the VLMT), *working memory* with the BACS Digit Sequencing

subtest (equivalent to the DST), *information processing* with the BACS Symbol Coding subtest (equivalent to the DSST), and *cognitive flexibility* using the time difference between TMT Part B and Part A.

Current antipsychotic treatment levels were was converted to chlorpromazine equivalent doses (CPZeq).[19] Further cohort characteristics from C1 and C2 along are available in the **eTable 1**. Lists of participants included in each of the different analyses can be found in the corresponding eTables.

Magnetic resonance imaging (MRI)

MRI scans in C1 were conducted with a 3.0 T MR scanner (Magnetom Skyra, Siemens Healthcare, Erlangen, Germany) equipped with a standard 20-channel phased-array head coil. MRI recordings in C2 were performed on a 3T Siemens MAGNETOM Prisma scanner (Siemens Healthineers AG) with a 32-channel head coil. T1-weighted scans were acquired by using a magnetization-prepared rapid acquisition gradient-echo sequence with an isotropic voxel size of 0.8 mm^3, 208 slices, a repetition time of 2500 ms, an echo time of 2.22 ms, a flip angle of 8, and a field of view of 256 mm^2.

MRI Preprocessing Pipeline

GMV was analyzed on a whole-brain voxel-wise (2 mm^3 voxel size) basis. The manual of the CAT12 toolbox (https://neuro-jena.github.io/cat12-help/) details the processing steps applied to the structural images. These steps consist of:

1) A 1st denoising step based on Spatially Adaptive Non-Local Means (SANLM) filtering.[20]
2) An Adaptive Maximum A Posteriori (AMAP) segmentation technique, which models local variations of intensity distributions as slowly varying spatial functions and thus achieves a homogeneous segmentation across cortical and subcortical structures.[25]
3) A 2nd denoising step using Markov Random Field approach which incorporates spatial prior information of adjacent voxels into the segmentation estimation generated by AMAP.[21]
4) A Local Adaptive Segmentation (LAS) step, which adjusts the images for white matter (WM) inhomogeneities and varying gray matter (GM) intensities caused by differing iron content in e.g., cortical, and subcortical structures. The LAS step is carried out before the final AMAP segmentation.
5) A Partial Volume Segmentation algorithm that is capable of modeling tissues with intensities between GM and WM, as well as GM and cerebrospinal fluid (CSF) and is applied to the AMAP-generated tissue segments.
6) A high-dimensional DARTEL registration of the image to an MNI-template generated from the MRI data of 555 healthy controls in the IXI database (http://brain-development.org/)

© 2026 Raabe FJ et al. *JAMA Psychiatry*.

MRI Data Quality Assurance

To assess homogeneity of the acquired MRI scans and assure a high standard of MRI data quality, we employed the homogeneity check option of the CAT12 toolbox. As part of the preprocessing, CAT12 calculates several individual quality measures for each MRI scan: NCR (Noise Contrast Ratio), ICR (Inhomogeneity Contrast Ratio) and RES (RMS resolution). These measures are combined into the weighted average image quality rating (IQR). The IQR measure is scaled from 0.5 to 10.5, where 0.5 is a 'perfect/excellent' score and 10.5 is deemed 'unacceptable/failed'. Values around 1 and 2 represent '(very) good' image quality, whereas values of 5 and higher indicate problematic images.[22] The data quality features were entered into the CAT12 "check homogeneity" module along with modulated (m) normalized (w) GM segments (p1). We then calculated the Mahalanobis distance between the mean correlation and weighted overall image quality. Mean correlation quantifies the homogeneity of all selected MRI data used for statistical analysis and is therefore a measure of image quality after pre-processing. The weighted overall image quality combines measurements of noise and spatial resolution of the images before pre-processing. Hence, calculating the Mahalanobis distance between these two measurements estimates image quality both before and after pre-processing. Following this approach, we only included cases with an overall image quality rating (IQR) of "good" to "very good". This led to the exclusion of one case, which deviated from the rest of the sample by more than two standard deviations. This protocol closely follows the general recommendation as given in the official CAT12-Manual (https://neuro-jena.github.io/cat12-help/). Since MRI data quality is influenced by the individual's age and often correlates with symptomatology,[23,24] we assumed a potential dimensional impact of data quality in our sample of mentally ill adolescents and young adults, even after passing the data quality assurance protocol. Since machine learning techniques such as sparse partial least squares (SPLS) can potentially detect and be driven by such subtle yet confounding factors, we included IQR as a feature into the analysis.

Electroencephalography (EEG)

Resting state EEG data were collected using 32 scalp electrodes and recorded with a BrainAmp amplifier (Brain Products, Martinsried, Germany). The setup had a sampling rate of 1000 Hz, using the Cz electrode as the reference. Electrode skin impedance was maintained below 5 kΩ. The electrodes were placed according to the International 10/20 system. The EEG recording session lasted a total of 10 minutes, divided into 5 minutes with eyes closed (eyes were opened for 3 seconds every 120 seconds to mitigate excessive alpha power). Throughout the session, participants were asked to stay calm and relaxed. An automated ICA preprocessing pipeline, adapted to Adams et al.[7] was utilized for preprocessing resting-state EEG with eyes closed condition, implemented in MATLAB (The Mathworks Inc.) using EEGLAB v2022.0 [25](https://sccn.ucsd.edu/eeglab/). First, the data was re-referenced to the mastoid bones (TP9 and TP10) and then downsampled to 256 Hz. A data filtering was applied, setting the frequency range between 1 to 70 Hz. rsEEG data were postprocessed in MNE

Python.[26] The canonical power spectrum density was calculated using the Welch Method, with 6-second windows and 3-second overlaps. (time_frequency.psd_array_welch). YASA library was used to extract absolute and relative power[27] of the different frequency bands: δ (1-4 Hz), θ (4-8 Hz), α (8-12 Hz), β (12-30 Hz), γ1 (30-50 Hz), γ2 (50-70 Hz).

Genotyping, quality control, imputation, and calculation of polygenic risk scores

Individuals were genotyped using Illumina's Global Screening Array (llumina Inc., San Diego, CA, USA). Illumina GenomeStudio v2.0.4 was used to process global screening array genotyping data and to obtain genotype calls for the study participants. Quality control (QC) was performed using PLINK v1.9/v2.[28] Single-nucleotide polymorphisms (SNPs) were excluded if they had a missing call rate greater than 2%, had a Minor Allele Frequency (MAF) less than 0.5%, or deviated from Hardy-Weinberg equilibrium with $p < 0.0001$. Individuals were excluded if they had a missing call rate greater than 2%, were duplicated samples according to the pairwise identity by state method, had a large deviation in their heterozygosity value (± 3.90 standard deviations), or had non-European ancestry according to a Multidimensional Scaling (MDS) analysis. MDS analysis was carried out with PLINK v1.9 to obtain a representation of genetic ancestry in our study, extracting the first 10 ancestry components. Palindromic SNPs and SNPs with a large MAF deviation (greater than 10%) with respect to 1000 Genomes European reference populations were also removed. Imputation was performed using the Haplotype Reference Consortium panel[29] in the Michigan Imputation Server.[30] A post-imputation QC was carried out to exclude SNPs that had an imputation quality score of R2 less than 0.3; or had a MAF less than 1%.

After genotype imputation, genotype dosage data were used to calculate schizophrenia polygenic risk scores (SCZ-PRS) for the N=277 genotyped individuals included in the analyzed cohort, based on the results of the Psychiatric Genomics Consortium wave 3 SCZ genome-wide association study.[31] Posterior single nucleotide polymorphism effect sizes were inferred under continuous shrinkage priors using PRS-CS.[32] The global shrinkage parameter (φ) was estimated using a fully Bayesian approach. The SCZ-PRS were eventually then calculated with PLINK v1.9[28] as the sum of risk alleles across SNPs carried by each individual multiplied by the inferred effect sizes.

iPSC culturing, neuronal differentiation

Differentiation of the NGN2-induced iPSC-derived excitatory pyramidal neurons (iN) on cohort level was carried out across two centers (Department of Psychiatry and Psychotherapy, University Hospital, LMU Munich, Germany, and Max Planck Institute of Psychiatry, Munich, Germany) as described previously.[33] In short: Six-well plates were coated with Geltrex (Geltrex in DMEM/F12) on Day 0 and left at 37°C for at least an hour. After incubation with Accutase for five minutes at 37°C, iPSCs were diluted 1:1 in DMEM/F12 supplemented with 1% FBS, centrifuged for five minutes at 300g, and then resuspended in StemMACS iPS Brew XF supplemented with Polybrene (6μg/ml) and 1x RevitaCell.

© 2026 Raabe FJ et al. *JAMA Psychiatry*.

Cells were counted and infected with pTet-O_Ngn2-puro and FUW-M2rtTA, and firstly they were incubated for 10 minutes at 37°C at a density of 1,000,000 cells/ml, and then seeded (25,000 cells/cm²) on the Matrigel-coated plates.

The following day (Day 1), media was changed with KSR media (KO DMEM, 15% Knockout Serum Replacement, 1x NEAA, 1x Glutamax, and 50 µM beta-mercaptoethanol).

To initiate neuronal patterning and directed differentiation, the media was supplemented with 10 µM SB431542, 2 µM XAV939, 0.1 µM LDN-193189, and 2 µg/ml doxycycline to initiate the directed neuronal differentiation with the overexpression of the transcription factor Neurogenin-2 (NGN2) in conjunction with small molecule application[34].

On day 2, media was replaced with a 1:1 mixture of KSR and N2 media (DMEM/F-12, 1x Glutamax, 3mg/ml glucose and 1x N-2), supplemented with 5 µM SB431542, 1 µM XAV939, 0.05 µM LDN-193189, 2 µg/ml doxycycline and 10 µg/ml puromycin, for selection purposes.

At day 3, media was changed with N2 media supplemented with 2 µg/ml doxycycline and 10µg/ml puromycin.

At day 4, neural progenitors were dissociated with Accutase for 5 minutes at 37°C. The cell suspension was diluted 1:1 with DMEM-F12 supplemented with 1%FBS and fully dissociated to a single-cell solution.

Cells were seeded at a density of 60.000 cells/cm² in NBM media (Neurobasal medium, 1x Glutamax, 1x NEAA, 1x B27 without vitamin A, 3 mg/ml glucose, 2% fetal bovine serum (FBS), freshly supplemented with 10 ng/ml BDNF, 10 ng/ml CNTF, 10 ng/ml GDNF, and 2 µg/ml doxycycline) on plates coated overnight with Poly-L- Ornithine (15 µg/ml) and then washed and coated with laminin (1 µg/ml) and Fibronectin (2 µg/ml) for at least 4h. At day 7, 5000/cm2 primary murine astrocytes were seeded on the iN culture. To prevent glial cell division and enrich post-mitotic neurons, NBM media was supplemented with 4 µM AraC on days 10 and 14.

With a density of 5000 mouse astrocytes/cm² on the induced iPSC-derived neurons (iNs), murine astrocytes were introduced by full media change on Day 7. To prevent glial cell division, 4 µM AraC was introduced to NBM medium on days 10 and 14. Afterwards, half media was removed changed every 3-4 days. From day 24 onwards, the cells were maintained in BrainPhys media (BrainPhys Neuronal Medium, 1x Glutamax, 1x NEAA, 1xB27 without vitamin A, 3 mg/ml glucose, 3% fetal calf serum (FCS), freshly supplemented with 10 ng/ml BDNF, 10 ng/ml CNTF, 10 ng/ml GDNF and 1µg/ml laminin) to promote neuronal maturation. iNs were collected at day 49 for endophenotyping.

Characterization and quality control of iPSC-derived neurons

To ensure the reliability and comparability of our iPSC-derived excitatory neuron (iN) model, we conducted extensive characterization and quality control, as reported in our previous study (see supplement).[6] In short, this quality control characterization included a genetic characterization and validation of the genomic stability of the generated iPSC lines, bulk RNA-Seq across all generated iN

lines and single-cell RNA-Seq profiling across selected donors, immunocytochemistry and electrophysiology (Extended Data Fig. 1–2 of [6]). For the statistical analysis, we used a linear mixed model accounting for the differentiation batch through a random effect. This extended cell line characterization confirmed that all iN cultures robustly expressed canonical excitatory neuronal markers, consistent with well-differentiated glutamatergic identity[6]. In silico deconvolution of bulk RNA-Seq data using single-cell-RNAseq (scRNAseq) of selected donors showed consistent cellular subtype composition across individuals and diagnostic groups, without significant shifts in differentiation outcomes between HC and SCZ donors[6]. Furthermore, electrophysiological recordings in a subset of iN donors revealed mature neuronal activity patterns in all analyzed lines that were largely comparable to mature primary human or mouse neurons[6]. For this study, we extended the cell culture characterization and have analyzed the TUBB3 and DAPI signal across all imaged plates to validate comparable culture conditions, comparing the total area of neurites and cell bodies across wells/fields of view (**eFigure 8B** and **8C)**. Moreover, based on RNAseq from the iN cultures, we characterized the maturity across groups, batch and centers of the iN culture based on previously published marker genes[35] (**eFigure 4C;** see also RNAseq analysis below). The added cell culture characterization confirmed and reproduced the maturation analysis from the original publication of the iPSC lines and demonstrate the absence of confounding batch- or diagnosis-driven heterogeneity in cell identity or maturation state and provide a foundation for the downstream molecular and phenotypic analyses reported in the current manuscript.

Imaging and synapse density analysis

Frequency of the pre-synaptic bouton marker Synapsin1 (SYN1) of electrophysiologically active iN-derived neuronal networks[6] was assessed as an approximate measure for the density of functional synapses. A subset of synapse density measurements using the same differentiation protocol was taken from our previous publication.[6] This dataset was extended for N=31 additional donors or donor replication using the following strategy: Analysis of Syn1 density was performed by confocal imaging using Leica DMi8 with Leica Application Suite X Software of iNs cultured on coverslips. Images were taken at 40X magnification and a resolution of 2048 dpi and exported as a TIFF file.

For each Field of View (FOV) ImageJ was used to identify the cell bodies on the TUBB3 channel, using the freehand selection tool. The TIFF files for each channel and the soma file were imported in CellProfiler 4.2.1 and analysis was carried out in a batch-specific manner. Nuclei were detected in the DAPI file by Adaptive Otsu three classes thresholding method for intensities (>0.006) and size (40-90 pixels). Afterwards, nuclei were filtered by texture and subsequently used together with the TUBB3 file to identify cell bodies using the Distance – B method (maximum distance from detected nuclei to TUBB3 signal: 40 pixels). The final cell body mask was generated by combining the manual and automatic detected soma.

© 2026 Raabe FJ et al. *JAMA Psychiatry.*

A partial neural network was identified using the Adaptative Minimum Cross-Entropy thresholding method (for intensities >0.01) in the TUBB3 mask. The cell body mask was subtracted from the partial neuronal network mask to generate the neurite mask.

Syn1 channel was adjusted with the "Speckle enhancement" function and subsequently used for identification of Syn1 speckles by Adaptive Sauvola method. Syn1 speckles were counted and quantified by occupied area (Syn1 area) in cell bodies and neurites, and normalized over the total cell body and neurite area. Next, Syn1 density measurements based on speckle area across were averaged across at least 8 FOV per well/each coverslip. Finally, the quantification of the immunocytochemistry signal of Syn1 density measurements were normalized by batch calculating a batch specific normalization factor. For this, we computed a normalization factor to account for differences in mean signal intensity. For that purpose, we computed the average synaptic density across all coverslips/wells in each batch. Subsequently, we normalized this average density to that observed in the first batch by dividing each mean batch density by the mean of batch 1. All results were merged with the previously published synaptic density measurements (see preprint form our group[6]) giving rise to a total of 107 distinct wells/coverslips across 4 differentiation batches and 42 different iPSC donors (**eTable 27**). The respective code can be found in the code repository of the paper (https://github.com/zillerlab).

To test for differences in Syn1 density between cases and controls, we performed linear mixed model analysis on Tukey's outlier test filtered and batch normalized synaptic density measurements with the following formula *Neurite_Syn1Area~ 1 +platform +(1|donor)+diagnosis*, where platform was either confocal imaging or CX5. To evaluate the potential presence of diagnosis specific differences in maturation level, neurite complexity or seeding density, we also included the total cell body area or the total neurite area as another fixed effect in the model, but did not observe any significant impact (cell body area p-value fixed effect: 0.348593, neurite area p= 0.801861). In addition, we also tested for diagnosis specific differences in neurite or cell body area using the same mixed model as for neurite Syn1 area, which also showed no significant difference (p= 0.858 and p= 0.826 respectively).

To perform EEG and synaptic density association and rpDCM analysis, Syn1 density based on Syn1 area in the neurite compartment was averaged across all wells/cover slips for each donor (**eTable 27**).

RNA-Seq data processing and analysis

Gene expression analysis was performed as described previously.[6] Briefly, paired-End raw RNA-Seq read sequences were aligned against the GRCh38 genome assembly with the GENCODE version 27 transcriptome definition using the STAR aligner (18) version 2.7.3a. Gene level read counts were obtained using the Rsubread package, collapsing exon level reads onto gene level features. Quantitative expression analysis was performed with DESeq2 version 1.22.2. Following size factor estimation, gene expression levels were normalized using the variance stabilizing transformation as implemented in DESeq2. These values were then used for further analysis. To characterize the maturation level, normalized marker genes were analyzed across a series of marker genes (Neural precursor genes:

© 2026 Raabe FJ et al. *JAMA Psychiatry*.

CDH1, HES1, PAX6, NOTCH1; early neuron marker genes: NEUROD1, DCX, CDH2, TUBB3; Marker genes of differentiated neurons: MAP2, MAPT, NEFH, BDNF; Pre-synaptic marker genes: SYN1, SYP, SNAP25; Post-synaptic marker genes: DLG4, CAMK2A, CAMK2B, DBN1; Glutamatergic marker genes: SLC17A7, SLC17A6; AMPAR marker genes: GRIA1, GRIA2, GRIA3, GRIA4; NMDAR marker genes: GRIN1, GRIN2A, GRIN2B, GRIN2C, GRIN2D)

Finally, we performed standard differential expression analysis testing for effect of diagnosis using differentiation site and sex as covariate. Genes showing an adjusted p-value ≤ 0.01 and a minimal absolute log2 fold-change ≥ 0.4 between Ctrl and SCZ were defined as DEGs and used as base set for all subsequent machine learning and gene expression imputation analyses.

Gene set enrichment analysis of DEPs

Gene set enrichment analysis was performed using the Enrichr tool,[36,37] testing differentially expressed genes (SCZ vs. HC) against the GO Biological Process 2025 library,[38-40] with all expressed genes in iPSC-derived neurons used as the background. Due to the exploratory nature of this analysis—and the relatively short DEG list, which is suboptimal for GSEAs in general—biological processes with nominal p-values < 0.05 were considered without adjustment for multiple comparisons. Visualization was performed using the Appyter volcano plot tool.[41] To further explore synapse-related functions, the DEG list was also analyzed using SynGO for curated synaptic annotation.[42]

Multi-marker Analysis of GenoMic Annotation (MAGMA) analysis of DEPs

To characterize the genetic background of the identified DEGs, formal gene enrichment analysis was performed using MAGMA (Multi-marker Analysis of GenoMic Annotation, version 1.09)[43] using the iN DEGs as genes of interest and the summary statistics of the latest schizophrenia GWAS.[44] After SNP-to-gene annotation (±10kb), gene-level analyses were carried out using the "SNPwise-mean" model. Gene-based p-values were obtained by combining the SNP p-values in a gene (±10kb) into a gene test statistic (sum of squared SNP Z-statistics) corrected for linkage disequilibrium between SNPs, gene size, and density.

Imputed gene expression

We used PriLer[45] train a machine learning model for each gene, predicting gene expression levels in iPSC-derived iNs from genotype only. We utilized a cohort of previously published matching genotype and RNA-Seq data from iPSC-derived pyramidal neurons from n=79 distinct donors to train individual elastic-net models for all empirically measured genes based on all single nucleotide polymorphisms (SNPs) within 200kb of the TSS of each gene as previously described. These models predict the variance in neuronal gene expression attributable to cis-acting genetic factors. We then applied these trained models to predict the cis-component of all genes with evidence for heritability of gene expression ($P \leq .10$ and $r \geq 0.1$ in the combined train and test-set) across the discovery and replication

© 2026 Raabe FJ et al. *JAMA Psychiatry*.

cohort. In order to also account for trans-acting genetic factors, we next established a support vector regression model to predict the gene expression levels of n=310 genes from both cis- and trans-factors. The latter genes were identified previously to be differentially expressed between SCZ and HC iPSCs-derived iNs (see previous paragraph; **eFigure 4F)**. To this end, we trained a support-vector regression model using the e1071 toolbox with a linear kernel, inferring the optimal hyperparameters using 5-fold cross validation. Prediction of the 310 candidate gene expression levels for all individuals with genotype included in this study was performed from all (cis-based) imputed transcriptome features, pre-filtering the genes for association with diagnosis status below a p-value of 0.1 to reduce the feature space. These analyses were performed independently for the discovery or replication cohorts (**eTable 1**). The predicted expression levels of these 310 genes showed good agreement with the empirical data (**eFigure 4D-H**) and were used as input features for subsequent prediction of brain/behavior score or EEG patterns.

Sparse partial least squares (SPLS) algorithm

SPLS overview

Multi-layered associations between clinical and cognitive phenotype and GMV were identified using the *sparse partial least squares* (SPLS) toolbox by Popovic et al.[46-48] SPLS uses singular-value decomposition to extract latent variables, i.e., multivariate signatures, that capture the shared covariance between two matrices - in our case, the cognitive phenotype matrix and the GMV matrix. Through a built-in elastic net, SPLS employs a powerful feature selection, while also handling multicollinearity, making the algorithm well-suited for uncovering the most relevant associations between high-dimensional data matrices with a high degree of interpretability. Moreover, SPLS uses projection deflation to iteratively remove the discovered associative effect from the matrices in order to look for further associations, thereby allowing the discovery of potentially multiple latent variables, i.e. multi-dimensional signatures within the same dataset. By projecting each individual's data onto the weight vectors of the latent variable (e.g. the '*schizophrenia neurocognition signature'*), individualized latent scores (e.g. *Cognition Score* and *Brain Score*) can be derived, providing individual loadings, i.e. expression, of these biobehavioral signatures in each individual. These latent scores are therefore individual representations of behavioral and structural phenotypes for further downstream analyses.

SPLS in details

The SPLS algorithm used in this analysis follows the original publication of Monteiro et al.[47] and has been described in a previous publication of our group.[46] Specifically, we used the open source toolbox by Popovic et al.[48] Like Partial Least Squares (PLS), SPLS requires two data matrices X and Y as inputs. In our study, X contains structural MRI data, while Y contains assessed cognitive domains and additional features (age, sex, years of education, study group status, IQ). n is the number of samples; p is the number of features in X and q is the number of features in Y. PLS provides insights into the brain's mechanisms

© 2026 Raabe FJ et al. *JAMA Psychiatry*.

by finding relationships between different measures (i.e., views) from the same participants, i.e., between neuroimaging and cognitive data, in a given population. PLS identifies a projection or latent space containing the relevant information in both views by finding pairs of weight vectors (generally called u and v) which maximize the covariance between the projections of the two views:[49]

1) $maximize_{\|u\|_2=\|v\|_2=1}\, Cov(Xu, Yv) = maximize_{\|u\|_2=\|v\|_2=1}\, u^T X^T Y v$

The weight vector pair is also called a latent variable (LV) as it explains one specific associative effect between the two different views. More specifically, the weight vectors place weights on each feature in the cognitive and the neuroimaging dataset, thus visualizing which features are associated with each other as well as the direction and the strength of this multivariate association. Hence, by studying this latent space, one can learn about the underlying relationship between cognitive information and brain measures.[47]

In contrast to regular PLS, SPLS enforces sparsity on the weight vectors u and v through hyperparameters c_u and c_v. c_u and c_v are the regularization hyperparameters that control the l_1-norm constraints of u and v, respectively. The l_1-norm constraints impose sparsity, which means that the lower the values of c_u and c_v are, the higher the sparsity in the respective view is.[50] This leads to the following optimization problem:

2) $maximize_{u,v}\, u^T X^T Y v \; subject\; to\; \|u\|_2^2 \leq 1, \|v\|_2^2 \leq 1, \|u\|_1 \leq c_u, \|v\|_1 \leq c_v$

Yet, this type of constraint can only select up to n features if $p > n$. Furthermore, it will remove features which might be relevant for the model but are correlated with other features which are already included. Zou and Hastie addressed this issue by adding the l_2-norm constraints.[51] For both l_1-norm and l_2-norm constraints to be active, the values of the hyperparameters must be between 1 and the square root of the number of features in the respective matrices. Therefore, the hyperparameter space is updated:

3) $1 \leq c_u \leq \sqrt{p}, 1 \leq c_v \leq \sqrt{q}$

Using the hyperparameter space of equation 3) and solving the optimization problem of equation 2) according to Witten et al.[50,52] leads to the following SPLS algorithm steps as described in Monteiro et al.[47]:

1. Let $C \leftarrow X^T Y$
2. Initialize v to have $\|v\|_2 = 1$
3. Repeat until convergence:
    a) Update u:
        i. $u \leftarrow Cv$
        ii. $u \leftarrow \frac{S(u, \Delta_u)}{\|S(u, \Delta_u)\|_2}$, where $\Delta_u = 0$ if this results in $\|u\|_1 \leq c_u$, otherwise Δ_u is set to be a positive constant such that $\|u\|_1 = c_u$
    b) Update v:
        i. $v \leftarrow C^T u$
        ii. $v \leftarrow \frac{S(v, \Delta_v)}{\|S(v, \Delta_v)\|_2}$, where $\Delta_v = 0$ if this results in $\|v\|_1 \leq c_v$, otherwise Δ_v is set

© 2026 Raabe FJ et al. *JAMA Psychiatry*.

to be a positive constant such that $\|v\|_1 = c_v$

4. If convergence is not reached after the iteration limit (default: 1000), return non-sparse weight vectors u and v

After a weight vector pair (h) is found by SPLS, its effect needs to be removed from the data, to look for the next possible weight vector pair ($h+1$). This process is called matrix deflation. In this setup, projection deflation is used as it has been shown to outperform the classic Hoteling's deflation, which is also used in Principal Component Analysis.[47,53,54] For matrices X and Y, the deflation process from iteration h to iteration $h+1$ is therefore computed as follows:

$$X_{h+1} \leftarrow X_h - (X_h u_h) u_h^T$$

$$Y_{h+1} \leftarrow Y_h - (Y_h v_h) v_h^T$$

The algorithm then uses the deflated matrices and looks for the next associative effect, i.e., the next LV. This way, SPLS iteratively provides LVs consisting of sparse weight vector pairs (u, v), uncovering several layers of associative effects within the dataset.

The second step of the SPLS algorithm involves the creation of latent scores. For every LV, weight vectors u and v are projected onto the matrixes X and Y, thus generating latent scores ε and ω.

$$\varepsilon_h = X u_h$$

$$\omega_h = Y v_h$$

These latent scores are finite numerical values, which represent the loading of each individual on these weight vectors, e.g., how high their GM probability in certain voxels is. Therefore, every individual can be represented within each LV space with its latent pheno and brain scores. These specific scores can then be used for post-hoc analyses to investigate the meaning and relevance of these individual loadings.

The models were generated and tested in a nested cross-validation (NCV) framework with 5 outer ($X2$, $Y2$) and 5 inner folds ($X1$, $Y1$). Individuals were stratified to the fold structure according to study group, so that all inner and outer folds contained an equal distribution of the three study groups (healthy controls (HC) with the subgroup of unaffected relatives (UR), and patients with schizophrenia (SCZ)) to avoid training on diagnosis-related effects. Within the inner folds, a 100x100 point grid search of both hyperparameters was conducted covering the entire hyperparameter space, in which both l_1- and l_2-norm constraints are fulfilled: $1 \leq c_u \leq \sqrt{p}$, $1 \leq c_v \leq \sqrt{q}$ (with p features in matrix X and q features in matrix Y). Lower c_u and c_v values lead to a sparser solution, whereas higher c_u and c_v values amount to a denser solution. At the upper limit, the maximum values of hyperparameters are: $c_u = \sqrt{p}$, $c_\text{v} = \sqrt{q}$. A SPLS analysis with c_u and c_v reaching these maximum values is equal to a regular PLS analysis, where every feature receives a weight and no feature is removed, i.e., no zero weights are given. Hence, our hyperparameter grid search includes the computation of one regular non-sparse PLS model (with c_u and c_v at the maximum limits) and an array of sparse PLS versions as lower c_u and c_v values are tested. Therefore, in this framework, the non-sparse regular PLS solution competes against the sparse PLS

solution in the hyperparameter optimization process. The weight vector pairs were generated using the training folds in the inner loop ($X1_{train}$, $Y1_{train}$):

$$(u, v) = spls(X1_{train}, Y1_{train}, c_u, c_v)$$

The model fit of the weight vector pair was then assessed by projecting them onto the testing folds ($X1_{test}$, $Y1_{test}$) in the inner loop and computing Spearman's correlation coefficient between the projections of the weight vectors u and v onto their respective data matrices $X1_{test}$ and $Y1_{test}$:

$$\rho = |Corr(X1_{test}u, Y1_{test}v)|$$

This approach delivers a simple and transparent measure of how well the weight vectors align the matrices to each other, i.e., how well they can maximize the covariance. The median correlation coefficient was computed for each hyperparameter combination in the inner loop. Afterwards, the best hyperparameter combinations ($\boldsymbol{c_{u-top}}, \boldsymbol{c_{v-top}}$) with the highest median correlation coefficients ($\boldsymbol{\rho_{top}}$) were retrained on the entirety of all 5 folds of the inner loop to increase the sample size for training once more:

$$\left(u_{top}, v_{top}\right) = spls(X2_{train}, Y2_{train}, c_{u-top}, c_{v-top})$$

The generalizability of the weight vector pairs ($\mathrm{u_{top}}, \mathrm{v_{top}}$) was tested by assessing the fit of their projections onto the previously held-out fold in the outer loop and thus computing the corresponding correlation coefficients (ρ_{max}).

$$\rho_{max} = \left|Corr\left(X2_{test}u_{opt}, Y2_{test}v_{opt}\right)\right|$$

Significance testing of this weight vector pair was achieved by permutation testing against B permutations. Within the fold structure of the outer loop, B permutated datasets were created by randomly reshuffling the order of participants in one matrix ($Yb2$) thus destroying relationship between the two matrices. The final model with the optimized hyperparameters (c_{u-opt}, c_{v-opt}) was then retrained and tested in each of the B permuted datasets, thus generating weight vectors u_b, v_b:

$$(u_b, v_b) = spls(X2_{train}, Yb2_{train}, c_{u-opt}, c_{v-opt})$$

$$\rho_b = |Corr(X2_{test}u_b, Y2_{test}v_b)|$$

Significance testing of the LV was done by assessing how often the model based on the permuted dataset performed better or equal to the model trained on the original dataset:

$$p = \frac{1 + \sum_{b=1}^{B} 1_{\rho_b \geq \rho_{max}}}{B + 1}$$

As our framework consisted of 5 outer folds, this approach led to 5 different models (i.e., 5 weight vector pairs u and v) for each LV iteration. Of these 5 different models, we selected the one model with the best performance as measured by means of permutation testing, i.e., the model that exhibited the lowest *P* value. If this optimal model passed significance testing against the FDR-corrected *P* value for multiple testing (5 models = 5 tests), the LV was deemed significant and the next LV was computed. This concept is known as the omnibus hypothesis, which was also applied in the original method paper of the SPLS algorithm.[47] The SPLS algorithm is an iterative process, in which based on hyperparameters

© 2026 Raabe FJ et al. *JAMA Psychiatry.*

c_u and c_v, the weight vectors u and v are computed in dependence of each other. First, u and v are initialized as non-sparse weight vectors based on regular singular value decomposition. Then an iterative process is set in motion, where first an enforcement of sparsity is attempted on weight vector u in dependence of weight vector v. Then sparsity is enforced on v, based on the previously computed weight vector u. This iterative process is repeated, where u and v are sequentially updated based on each other's previous modification until convergence between the vectors is reached. Hence, every hyperparameter setup c_u and c_v leads to a unique process of finding converging weight vectors u and v that were generated in a dialectic manner. Thus, the multivariate information is contained in this highly specific combination of weight vectors u and v, with both vectors containing mathematical information of the other. This, in turn, makes weight vectors u and v from different models, such as in our 5x5 fold NCV, not suitable for usual merging techniques (weighted mean/mean/median merging or majority voting) as every vector u is dependent on the corresponding vector v. Therefore, we used the omnibus hypothesis to determine our final LV model out of the 5 computed within the NCV structure of each LV iteration. Using our 5x5-fold outer and inner cross-validation loops can lead to high variance in the results. After training on the inner loops and then testing on the outer loops, 5 models with 5 *P* values are obtained. A criterion is then needed to determine whether any statistically significant effects were indeed found. For this, we used the omnibus hypothesis, where a statistical test is performed *j*-times to test a null-hypothesis H_j. Following the omnibus approach, the combined hypothesis H_R over all tests j is: "All the hypothesis H_j are true". This hypothesis will be rejected if any of the H_j hypothesis is rejected.[55] In our specific case, the omnibus hypothesis states that if any of the 5 *P* values (obtained in the 5 outer folds) is statistically significant (corrected for multiple testing *j*-times), then the omnibus hypothesis will be rejected, and the detected effect will be deemed significant. Therefore, the omnibus hypothesis will be rejected if any of the 5 splits generates a *P* value below .05 (adjusted for multiple testing). Of all significant splits, the model with the lowest *P* value will be determined as the final LV model.[47] The computation ends as soon as none of the 5 splits of the LV iteration did not pass the test for significance, which renders the entire LV not significant. Since deflating the data matrices of non-significant effects would be not justified, the analysis pipeline stops after the first non-significant LV was detected.

Full SPLS analysis was performed on C1 (*MIMICSS*) as discovery sample to identify LVs.

For replication of the SPLS signatures in the C2 (*CDP*), propensity score matching was employed first.[56,57] Specifically, a general linear model with a binomial distribution was fitted using the matching features age, sex and study group (HC, UR, SCZ) to predict cohort assignment (*MIMICSS, CDP*). After that, individuals from C2 were drawn based on k-nearest neighbor computation (k=7) to retrieve a subsample of C2 individuals that most closely matched the C1 cohort with regards to the matching features. Replication analysis was performed by projecting the data of the C2 (*CDP*) sample onto the LVs retrieved

in the C1 (*MIMICSS*) sample and computing the correlation coefficient between the resulting latent scores. Covariate correction and sites remained the same in the C2 sample.

Support Vector Regression (SVR)

The entire machine learning analysis was conducted on the NeuroMiner machine learning platform,[58] release version 1.3 (https://github.com/neurominer-git/NeuroMiner_1.3).

Repeated nested cross-validation (RNCV): We embedded the algorithm in a repeated nested cross-validation (CV) framework with 10 folds and 10 permutations on the inner (CV1) and outer (CV2) cycle to prevent overfitting and information leaking, while enhancing generalizability.[59] Hyperparameter optimization was performed on the CV1 cycle, while model testing was done on the CV2 cycle.[60] Thus, all steps involving group-level statistical procedures (e.g., scaling, pruning, covariate correction) took place in the CV1 cycle. We extended the NCV to a repeated NCV approach[59] at both the CV2 and the CV1 level by randomly permuting the individuals within their groups and repeating the CV cycle for each of these permutations to avoid batch effects and, again, increase generalizability.

Preprocessing: Each feature was scaled, pruned and, if necessary, corrected for covariates within the NCV scheme.[61,62] Only EEG data, whether used as predictors or labels, was corrected for age and sex based on betas computed within the HC subpopulation. No other data was corrected for covariates.

Model testing: After training and optimization in the CV1 level, the resulting models were applied to the corresponding CV2 fold by preprocessing the best discriminative variables using the learned scaling from the CV1 cycle and determining each testing individual's label through majority voting across all ensemble models. In other words, in each variable evaluation step in the CV1 level, the SVR algorithm modeled linear relationships between features and labels. Due to the repeated nested cross-validation framework, an ensemble of 100 models (n=10 repetition x k=10 folds) for each CV2 partition ("CV1 ensemble") was created. Furthermore, due to the 10 repetitions of the CV2 cycle, we were able to establish a final out-of-training label prediction for a given individual by combining all CV1 ensembles into a larger CV2 ensemble, in which the given individual had not served for model training and optimization at the CV1 level. This ensemble generation procedure has been described previously[63] and is a feature of the model generation and validation process implemented in NeuroMiner.

Model significance: To assess statistical significance of the SVR models, we employed permutation testing.[64] We performed 5000 random permutations of the outcome labels. For each permutation, we retrained the model in the repeated NCV structure using the respective feature subsets obtained from the observed-label analyses. For each permutation, we accumulated the predictions of the random models into a permuted ensemble prediction for each CV2 subject. Thus, we built a null distribution of out-of-training regression performance (MAE) and then calculated the significance of the observed out-of-training MAE as the number of events where the permuted out-of-training MAE was higher or equal

© 2026 Raabe FJ et al. *JAMA Psychiatry*.

to the observed MAE divided by the number of permutations performed. The significance of the model was determined at q=0.05.

The SVR models were applied onto the replication dataset using the same preprocessing pipeline as in the discovery sample. Specifically, the replication data was stratified to the identical CV structure used for model generation and the predictions were pooled across the CV structure to retrieve a cross-validated performance of the SVR model in the replication sample. No further retraining was conducted within the CV structure.

Detailed SVR Pipeline

In detail, the machine learning pipeline consisted of the following steps:

Repeated nested cross-validation

- CV1 cycle: 10 folds and 10 permutations
- CV2 cycle: 10 folds and 10 permutations

Preprocessing Setup

1. Feature-wise Scaling from 0 to 1, zeroing-out completely non-finite features.
2. Pruning of non-informative features with zero variance.
3. Imputation within in matrix block using the median of 7 nearest neighbors (k-nearest-neighbor imputation).
4. Only for EEG as predictor data: attenuating covariate effects (age, sex) via partial correlations. Beta values computed within the HC subpopulation and then used for correction within the entire sample.
5. Feature-wise Scaling from 0 to 1, zeroing-out completely non-finite features.

Machine Learning Algorithm Parameter Setup

1. ML model performance criterion: Mean Absolute Error (MAE)
2. Target scaling: [0, 1]
3. Selection and Configuration of ML algorithm
    a. LIBSVM 3.1.2 with instance weighting support
    b. Regressor type: epsilon-SVR
    c. Kernel type: linear
    d. Cache size: 500 MB
    e. Termination criterion: 0.001
    f. Shrinking heuristics: enabled

Model Optimization Parameters

1. Slack/Regularization parameters: [0.015625, 0.03125, 0.0625, 0.125, 0.25, 0.5, 1]
2. Eps-SVR parameters: [0.05, 0.075, 0.1, 0.125, 0.15, 0.2]
3. No regularization of model selection
4. Cross-parameter model selection process: Single optimum model (no ensemble)

Visualization of predictive pattern elements

We used two computational approaches to visualize the predictive patterns of the SVR models. The detailed mathematical background of these procedures has already been described in the Supplementary Material of Koutsouleris et al.[65] First, sign-based consistency mapping was employed to measure predictive stability. It is based on an approach proposed by Gómez-Verdejo et al.[66] toward wrapper-based feature selection strategies. This method assesses how consistently a feature was weighted either positively or negatively across all cross-validated models. In doing so, a *P* value can be computed, measuring whether the distribution of positive and negative weightings of a certain feature across all computed models is significantly different from a random null distribution. The *P* value therefore indicates whether a certain feature received predominantly positive or negative weights above chance level. Thus, sign-based consistency mapping allows us to identify those features, which have been significantly predictive for higher or lower label values. For those features that passed the sign-based consistency threshold and were therefore stable, significant predictors, we calculated the cross-validation ratio (CVR), by computing the grand mean and standard error of all SVR weight vectors across all computed models. The CVR as a measure for the extent of which a pattern was weighted towards one of the labels. It is inspired by the bootstrap ratio commonly used in the Partial Least Squares literature and described in Krishnan et al.[67] Thus, the CVR indicates how predictive a certain feature was for higher or lower label values. While the direction of the CVR (e.g., positive, or negative) indicates the direction of the label prediction, the absolute value of the CVR indicates how strong the contribution of the feature was for the overall prediction. Combining these two approaches allowed us to identify the most stable and most decisive predictors for our models, therefore giving way to further assessment and interpretation.

Software

All analyses were conducted using MATLAB R2022a (MathWorks, Natick, MA, USA). Specifically, we used functions from the MATLAB Statistics and Machine Learning Toolbox as well as the machine learning platform NeuroMiner, release version 1.3, developed by Nikolaos Koutsouleris (https://github.com/neurominer-git/NeuroMiner_1.3).[58,68]

Reverse personalized dynamic causal modelling (rpDCM)

For the reverse personalized dynamic causal model (rpDCM), we adapted a previously established canonical microcircuit model,[7] representing a biophysical model of interacting pyramidal, interneuron, and spiny stellate neuronal cell populations (**Figure 5A)**. This model simulates neuronal mass and resulting circuit activity, giving rise to a simulated EEG power spectrum. The model can be parameterized with different neuronal connectivity settings and strength within and between the various neuronal populations to assess the impact of these parameters on the EEG. We parameterized the model by the synaptic strength (synaptic gain) of all excitatory synapses in the microcircuit and simulated the resting state EEG pattern as a function of different overall synaptic strength levels (**eFigure 9**).

© 2026 Raabe FJ et al. *JAMA Psychiatry*.

Subsequently, utilized our empirical synaptic density measurements in iNs obtained from 42 SCZ and HC donors (**Figure 5B,** and **5C; eFigure 8A-C**), normalized them to the synaptic gain parameter interval of the DCM ([0.7, 1.0]) to obtain personalized rpDCMs for each SCZ patient and HC. Subsequently, we simulated the respective relative EEG power spectrum of each individual (**Figure 5D)** and compared the absolute power for each frequency range to the power of the eyes closed, resting state EEG for each individual and electrode for 28 individuals. The code for the DCM model and analysis can be found at: https://github.com/zillerlab.

Statistical analysis

Group comparisons and association analyses (incl. covariate analyses) were performed using linear (mixed) or logistic regression models as indicated in the respective sections. Results were corrected for multiple testing using q-value as implemented in the respective R package[69] where appropriate and described in the respective analysis.

© 2026 Raabe FJ et al. *JAMA Psychiatry*.

eReferences.

1. Fan L, Li H, Zhuo J, et al. The Human Brainnetome Atlas: A New Brain Atlas Based on Connectional Architecture. *Cereb Cortex*. Aug 2016;26(8):3508-26. doi:10.1093/cercor/bhw157
2. Diedrichsen J, Balsters JH, Flavell J, Cussans E, Ramnani N. A probabilistic MR atlas of the human cerebellum. *NeuroImage*. 2009/05/15/ 2009;46(1):39-46.
3. Yeo BT, Krienen FM, Sepulcre J, et al. The organization of the human cerebral cortex estimated by intrinsic functional connectivity. *J Neurophysiol*. Sep 2011;106(3):1125-65. doi:10.1152/jn.00338.2011
4. Buckner RL, Krienen FM, Castellanos A, Diaz JC, Yeo BT. The organization of the human cerebellum estimated by intrinsic functional connectivity. *J Neurophysiol*. Nov 2011;106(5):2322-45. doi:10.1152/jn.00339.2011
5. Trubetskoy V, Pardiñas AF, Qi T, et al. Mapping genomic loci implicates genes and synaptic biology in schizophrenia. *Nature*. 2022/04/01 2022;604(7906):502-508. doi:10.1038/s41586-022-04434-5
6. Raabe FJ, Hausruckinger A, Gagliardi M, et al. Polygenic risk for schizophrenia converges on alternative polyadenylation as molecular mechanism underlying synaptic impairment. *bioRxiv*. 2024;doi:10.1101/2024.01.09.574815
7. Adams RA, Pinotsis D, Tsirlis K, et al. Computational Modeling of Electroencephalography and Functional Magnetic Resonance Imaging Paradigms Indicates a Consistent Loss of Pyramidal Cell Synaptic Gain in Schizophrenia. *Biol Psychiatry*. Jan 15 2022;91(2):202-215. doi:10.1016/j.biopsych.2021.07.024
8. Budde M, Anderson-Schmidt H, Gade K, et al. A longitudinal approach to biological psychiatric research: The PsyCourse study. *Am J Med Genet B Neuropsychiatr Genet*. Aug 2 2018;doi:10.1002/ajmg.b.32639
9. Krcmar L, Jager I, Boudriot E, et al. The multimodal Munich Clinical Deep Phenotyping study to bridge the translational gap in severe mental illness treatment research. *Front Psychiatry*. 2023;14:1179811. doi:10.3389/fpsyt.2023.1179811
10. Sheehan DV, Lecrubier Y, Sheehan KH, et al. The Mini-International Neuropsychiatric Interview (M.I.N.I.): the development and validation of a structured diagnostic psychiatric interview for DSM-IV and ICD-10. *J Clin Psychiatry*. 1998;59 Suppl 20:22-33;quiz 34-57.
11. Kalman JL, Burkhardt G, Adorjan K, et al. Biobanking in everyday clinical practice in psychiatry—The Munich Mental Health Biobank. Original Research. *Frontiers in Psychiatry*. 2022-July-22 2022;13doi:10.3389/fpsyt.2022.934640
12. Kay SR, Fiszbein A, Opler LA. The Positive and Negative Syndrome Scale (PANSS) for Schizophrenia. *Schizophrenia Bulletin*. 1987;13(2):261-276. doi:10.1093/schbul/13.2.261
13. Helmstaedter C, Durwen H. VLMT: Verbaler Lern-und Merkfähigkeitstest: Ein praktikables und differenziertes Instrumentarium zur Prüfung der verbalen Gedächtnisleistungen. *Schweizer Archiv für Neurologie, Neurochirurgie und Psychiatrie*. 1990;
14. Müller H, Hasse-Sander I, Horn R, Helmstaedter C, Elger CE. Rey Auditory-Verbal Learning Test: structure of a modified German version. *J Clin Psychol*. Nov 1997;53(7):663-71. doi:10.1002/(sici)1097-4679(199711)53:7<663::aid-jclp4>3.0.co;2-j
15. Tewes U. *HAWIE-R: Hamburg-Wechsler-Intelligenztest für Erwachsene, Revision 1991 ; Handbuch und Testanweisung*. Huber; 1994.
16. Tombaugh T. Trail Making Test A and B: Normative data stratified by age and education. *Archives of Clinical Neuropsychology*. 2004;19(2):203-214. doi:10.1016/s0887-6177(03)00039-8
17. Schmidt K-H, Metzler P. *WST: Wortschatztest*. Beltz Test GmbH; 1992.
18. Keefe RSE, Goldberg TE, Harvey PD, Gold JM, Poe MP, Coughenour L. The Brief Assessment of Cognition in Schizophrenia: reliability, sensitivity, and comparison with a standard neurocognitive battery. *Schizophrenia Research*. 2004/06/01/ 2004;68(2):283-297. doi:10.1016/j.schres.2003.09.011
19. Leucht S, Samara M, Heres S, Davis JM. Dose Equivalents for Antipsychotic Drugs: The DDD Method. *Schizophr Bull*. Jul 2016;42 Suppl 1:S90-4. doi:10.1093/schbul/sbv167
20. Manjon JV, Tohka J, Garcia-Marti G, et al. Robust MRI brain tissue parameter estimation by multistage outlier rejection. *Magn Reson Med*. Apr 2008;59(4):866-73. doi:10.1002/mrm.21521

© 2026 Raabe FJ et al. *JAMA Psychiatry*.

21. Rajapakse JC, Giedd JN, Rapoport JL. Statistical approach to segmentation of single-channel cerebral MR images. *IEEE Trans Med Imaging*. Apr 1997;16(2):176-86. doi:10.1109/42.563663
22. Collins DL, Zijdenbos AP, Kollokian V, et al. Design and construction of a realistic digital brain phantom. *IEEE Trans Med Imaging*. Jun 1998;17(3):463-8. doi:10.1109/42.712135
23. Pardoe HR, Kucharsky Hiess R, Kuzniecky R. Motion and morphometry in clinical and nonclinical populations. *Neuroimage*. Jul 15 2016;135:177-85. doi:10.1016/j.neuroimage.2016.05.005
24. Savalia NK, Agres PF, Chan MY, Feczko EJ, Kennedy KM, Wig GS. Motion-related artifacts in structural brain images revealed with independent estimates of in-scanner head motion. *Hum Brain Mapp*. Jan 2017;38(1):472-492. doi:10.1002/hbm.23397
25. Delorme A, Makeig S. EEGLAB: an open source toolbox for analysis of single-trial EEG dynamics including independent component analysis. *Journal of Neuroscience Methods*. 2004/03/15/ 2004;134(1):9-21. doi:https://doi.org/10.1016/j.jneumeth.2003.10.009
26. Gramfort A, Luessi M, Larson E, et al. MEG and EEG data analysis with MNE-Python. (1662-4548 (Print))
27. Vallat RA-O, Walker MA-O. An open-source, high-performance tool for automated sleep staging. LID - 10.7554/eLife.70092 [doi] LID - e70092. (2050-084X (Electronic))
28. Chang CC, Chow CC, Tellier LC, Vattikuti S, Purcell SM, Lee JJ. Second-generation PLINK: rising to the challenge of larger and richer datasets. *Gigascience*. 2015;4:7. doi:10.1186/s13742-015-0047-8
29. McCarthy S, Das S, Kretzschmar W, et al. A reference panel of 64,976 haplotypes for genotype imputation. *Nat Genet*. Oct 2016;48(10):1279-83. doi:10.1038/ng.3643
30. Das S, Forer L, Schonherr S, et al. Next-generation genotype imputation service and methods. *Nat Genet*. Oct 2016;48(10):1284-1287. doi:10.1038/ng.3656
31. Trubetskoy V, Pardinas AF, Qi T, et al. Mapping genomic loci implicates genes and synaptic biology in schizophrenia. *Nature*. Apr 2022;604(7906):502-508. doi:10.1038/s41586-022-04434-5
32. Ge T, Chen CY, Ni Y, Feng YA, Smoller JW. Polygenic prediction via Bayesian regression and continuous shrinkage priors. *Nat Commun*. Apr 16 2019;10(1):1776. doi:10.1038/s41467-019-09718-5
33. Rummel CK, Gagliardi M, Ahmad R, et al. Massively parallel functional dissection of schizophrenia-associated noncoding genetic variants. *Cell*. 2023;doi:10.1016/j.cell.2023.09.015
34. Nehme R, Zuccaro E, Ghosh SD, et al. Combining NGN2 Programming with Developmental Patterning Generates Human Excitatory Neurons with NMDAR-Mediated Synaptic Transmission. *Cell Rep*. May 22 2018;23(8):2509-2523. doi:10.1016/j.celrep.2018.04.066
35. Lin W, Shiomoto S, Yamada S, et al. Dendritic spine formation and synapse maturation in transcription factor-induced human iPSC-derived neurons. *iScience*. Apr 21 2023;26(4):106285. doi:10.1016/j.isci.2023.106285
36. Chen EY, Tan CM, Kou Y, et al. Enrichr: interactive and collaborative HTML5 gene list enrichment analysis tool. *BMC Bioinformatics*. Apr 15 2013;14(1471-2105 (Electronic)):128. doi:10.1186/1471-2105-14-128
37. Kuleshov MV, Jones MR, Rouillard AD, et al. Enrichr: a comprehensive gene set enrichment analysis web server 2016 update. *Nucleic Acids Res*. Jul 8 2016;44(W1):W90-7. doi:10.1093/nar/gkw377
38. Consortium TGO, Aleksander SA, Balhoff J, et al. The Gene Ontology knowledgebase in 2023. *Genetics*. 2023;224(1)doi:10.1093/genetics/iyad031
39. Ashburner M, Ball CA, Blake JA, et al. Gene ontology: tool for the unification of biology. The Gene Ontology Consortium. *Nat Genet*. May 2000;25(1):25-9. doi:10.1038/75556
40. Aleksander SA, Balhoff J, Carbon S, et al. The Gene Ontology knowledgebase in 2023. *Genetics*. May 4 2023;224(1)doi:10.1093/genetics/iyad031
41. Clarke DJB, Jeon M, Stein DJ, et al. Appyters: Turning Jupyter Notebooks into data-driven web apps. *Patterns*. 2021;2(3)doi:10.1016/j.patter.2021.100213
42. Koopmans F, van Nierop P, Andres-Alonso M, et al. SynGO: An Evidence-Based, Expert-Curated Knowledge Base for the Synapse. *Neuron*. 2019;103(2):217-234.e4. doi:10.1016/j.neuron.2019.05.002
43. de Leeuw CA, Mooij JM, Heskes T, Posthuma D. MAGMA: generalized gene-set analysis of GWAS data. *PLoS Comput Biol*. Apr 2015;11(4):e1004219. doi:10.1371/journal.pcbi.1004219

© 2026 Raabe FJ et al. *JAMA Psychiatry*.

44. Trubetskoy V, Pardiñas AF, Qi T, et al. Mapping genomic loci implicates genes and synaptic biology in schizophrenia. *Nature*. Apr 2022;604(7906):502-508. doi:10.1038/s41586-022-04434-5
45. Trastulla L, Dolgalev G, Moser S, et al. Distinct genetic liability profiles define clinically relevant patient strata across common diseases. *Nat Commun*. Jul 1 2024;15(1):5534. doi:10.1038/s41467-024-49338-2
46. Popovic D, Ruef A, Dwyer DB, et al. Traces of Trauma: A Multivariate Pattern Analysis of Childhood Trauma, Brain Structure, and Clinical Phenotypes. *Biol Psychiatry*. Dec 1 2020;88(11):829-842. doi:10.1016/j.biopsych.2020.05.020
47. Monteiro JM, Rao A, Shawe-Taylor J, Mourão-Miranda J. A multiple hold-out framework for Sparse Partial Least Squares. *J Neurosci Methods*. Sep 15 2016;271:182-94. doi:10.1016/j.jneumeth.2016.06.011
48. *SPLS Toolbox*. GitHub; 2022. https://github.molgen.mpg.de/DavidPopovic/SPLS_Toolbox_2022
49. Wegelin J. A Survey of Partial Least Squares (PLS) Methods, with Emphasis on the Two-Block Case. *Technical report*. 04/10 2000;
50. Witten DM, Tibshirani R, Hastie T. A penalized matrix decomposition, with applications to sparse principal components and canonical correlation analysis. *Biostatistics*. Jul 2009;10(3):515-34. doi:10.1093/biostatistics/kxp008
51. Zou H, Hastie T. Regularization and Variable Selection Via the Elastic Net. *Journal of the Royal Statistical Society Series B: Statistical Methodology*. 2005;67(2):301-320. doi:10.1111/j.1467-9868.2005.00503.x
52. Witten DM, Tibshirani RJ. Extensions of sparse canonical correlation analysis with applications to genomic data. *Stat Appl Genet Mol Biol*. 2009;8(1):Article28. doi:10.2202/1544-6115.1470
53. Monteiro JM, Rao A, Ashburner J, Shawe-Taylor J, Mourão-Miranda J. Leveraging Clinical Data to Enhance Localization of Brain Atrophy. Springer International Publishing; 2016:60-68.
54. Mackey L. *Deflation Methods for Sparse PCA*. vol 21. 2008:1017-1024.
55. Nichols TE, Holmes AP. Nonparametric permutation tests for functional neuroimaging: a primer with examples. *Hum Brain Mapp*. Jan 2002;15(1):1-25. doi:10.1002/hbm.1058
56. Ho DE, Imai K, King G, Stuart EA. Matching as nonparametric preprocessing for reducing model dependence in parametric causal inference. *Polit Anal*. Sum 2007;15(3):199-236. doi:10.1093/pan/mpl013
57. Ho DE, Imai K, King G, Stuart EA. MatchIt: Nonparametric Preprocessing for Parametric Causal Inference. *Journal of Statistical Software*. Jun 2011;42(8)
58. Koutsouleris N, Kahn RS, Chekroud AM, et al. Multisite prediction of 4-week and 52-week treatment outcomes in patients with first-episode psychosis: a machine learning approach. *Lancet Psychiatry*. Oct 2016;3(10):935-946. doi:10.1016/s2215-0366(16)30171-7
59. Dwyer DB, Falkai P, Koutsouleris N. Machine Learning Approaches for Clinical Psychology and Psychiatry. *Annu Rev Clin Psychol*. May 7 2018;14:91-118. doi:10.1146/annurev-clinpsy-032816-045037
60. Ruschhaupt M, Huber W, Poustka A, Mansmann U. A compendium to ensure computational reproducibility in high-dimensional classification tasks. *Stat Appl Genet Mol Biol*. 2004;3:Article37. doi:10.2202/1544-6115.1078
61. Dukart J, Schroeter ML, Mueller K, Alzheimer's Disease Neuroimaging I. Age correction in dementia--matching to a healthy brain. *PLoS One*. 2011;6(7):e22193. doi:10.1371/journal.pone.0022193
62. Koutsouleris N, Meisenzahl EM, Borgwardt S, et al. Individualized differential diagnosis of schizophrenia and mood disorders using neuroanatomical biomarkers. *Brain*. Jul 2015;138(Pt 7):2059-73. doi:10.1093/brain/awv111
63. Koutsouleris N, Riecher-Rössler A, Meisenzahl EM, et al. Detecting the psychosis prodrome across high-risk populations using neuroanatomical biomarkers. *Schizophr Bull*. Mar 2015;41(2):471-82. doi:10.1093/schbul/sbu078
64. Golland P, Fischl B. Permutation tests for classification: towards statistical significance in image-based studies. *Inf Process Med Imaging*. Jul 2003;18:330-41. doi:10.1007/978-3-540-45087-0_28

© 2026 Raabe FJ et al. *JAMA Psychiatry*.

65. Koutsouleris N, Dwyer DB, Degenhardt F, et al. Multimodal Machine Learning Workflows for Prediction of Psychosis in Patients With Clinical High-Risk Syndromes and Recent-Onset Depression. *JAMA Psychiatry*. Feb 1 2021;78(2):195-209. doi:10.1001/jamapsychiatry.2020.3604
66. Gómez-Verdejo V, Parrado-Hernández E, Tohka J. Sign-Consistency Based Variable Importance for Machine Learning in Brain Imaging. *Neuroinformatics*. Oct 2019;17(4):593-609. doi:10.1007/s12021-019-9415-3
67. Krishnan A, Williams LJ, McIntosh AR, Abdi H. Partial Least Squares (PLS) methods for neuroimaging: a tutorial and review. *Neuroimage*. May 15 2011;56(2):455-75. doi:10.1016/j.neuroimage.2010.07.034
68. Koutsouleris N, Kambeitz-Ilankovic L, Ruhrmann S, et al. Prediction Models of Functional Outcomes for Individuals in the Clinical High-Risk State for Psychosis or With Recent-Onset Depression: A Multimodal, Multisite Machine Learning Analysis. *JAMA Psychiatry*. 2018;75(11):1156-1172. doi:10.1001/jamapsychiatry.2018.2165
69. Storey JD. The positive false discovery rate: A Bayesian interpretation and the q-value. *Ann Stat*. Dec 2003;31(6):2013-2035. doi:DOI 10.1214/aos/1074290335

© 2026 Raabe FJ et al. *JAMA Psychiatry*.
