# Supplementary material for: Bridging the Scales via Personalized Cellular Modeling and Deep Phenotyping in Schizophrenia
Source: JAMA Psychiatry. 2026 Mar 28;83(5):510–22. doi: 10.1001/jamapsychiatry.2026.0576 (PMC13033174; doi:10.1001/jamapsychiatry.2026.0576)
Supplement: Supplement 3. — CDP Working Group [file jamapsychiatry-e260576-s003.pdf]

*Group Name(s): CDP Working Group							
***First Name and Middle Initial(s)**	***Last Name**	***Suffix (eg, Jr, III)**	Academic Degrees	Institution	Location (city, state/province, country)	Role or Contribution, eg, chair, principal investigator	Group (if more than 1 Group listed in the byline) and/or Subgroup (eg, Steering Committee)
Stephanie	Behrens			Department of Psychiatry and Psychotherapy, LMU University Ho		Sample preparation an	CDP Working Group
Emanuel	Boudriot			Department of Psychiatry and Psychotherapy, LMU University Ho		Clinical data collection	CDP Working Group
Man-Hsin	Chang			Department of Psychiatry and Psychotherapy, LMU University Ho		Sample preparation an	CDP Working Group
Valéria	de Almeida			Department of Psychiatry and Psychotherapy, LMU University Ho		Sample preparation an	CDP Working Group
Sylvia	de Jonge			Department of Psychiatry and Psychotherapy, LMU University Ho		Sample preparation an	CDP Working Group
Fanny	Dengl			Department of Psychiatry and Psychotherapy, LMU University Ho		Clinical data collection	CDP Working Group
Lina	Dürrwald			Max Planck Institute of Psychiatry, 80804 Munich, Germany		Sample preparation an	CDP Working Group
Peter	Falkai			Department of Psychiatry and Psychotherapy, LMU University Ho		Supervisor	CDP Working Group
Laura E.	Fischer			Max Planck Institute of Psychiatry, 80804 Munich, Germany		Sample preparation an	CDP Working Group
Nadja	Gabellini			Department of Psychiatry and Psychotherapy, LMU University Ho		Sample preparation an	CDP Working Group
Vanessa	Gabriel			Department of Psychiatry and Psychotherapy, LMU University Ho		Clinical data collection	CDP Working Group
Sabrina	Galinski			Department of Psychiatry and Psychotherapy, LMU University Ho		Sample preparation an	CDP Working Group
Thomas	Geyer			Department of Psychology, Ludwig Maximilian University of Mur		Supervisor	CDP Working Group
Katharina	Hanken			Department of Psychiatry and Psychotherapy, LMU University Ho		Clinical data collection	CDP Working Group
Alkomiet	Hasan			Department of Psychiatry, Psychotherapy, and Psychosomatics, I		Supervisor	CDP Working Group
Genc	Hasanaj			Department of Psychiatry and Psychotherapy, LMU University Ho		Sample preparation an	CDP Working Group
Alexandra	Hisch			Department of Psychiatry and Psychotherapy, LMU University Ho		Clinical data collection	CDP Working Group
Georgios	Ioannou			Department of Psychiatry and Psychotherapy, LMU University Ho		Clinical data collection	CDP Working Group
Marcus	Ising			Max Planck Institute of Psychiatry, 80804 Munich, Germany		Sample preparation an	CDP Working Group
Iris	Jäger			Evidence-based psychiatry and psychotherapy, Faculty of Medici		Clinical data collection	CDP Working Group
Tengjia	Jiang			Max Planck Institute of Psychiatry, 80804 Munich, Germany		Data processing	CDP Working Group
Marcel	Kallweit			Department of Psychiatry and Psychotherapy, LMU University Ho		Clinical data collection	CDP Working Group
Temmuz	Karali			Department of Psychiatry and Psychotherapy, LMU University Ho		Data processing	CDP Working Group
Susanne	Karch			Department of Psychiatry and Psychotherapy, LMU University Ho		Supervisor	CDP Working Group
Berkhan	Karslı			Department of Psychiatry and Psychotherapy, LMU University Ho		Data processing	CDP Working Group
Daniel	Keeser			Department of Psychiatry and Psychotherapy, LMU University Ho		Principal Investigator	CDP Working Group
Christoph	Kern			Department of Ophthalmology, LMU University Hospital, LMU M		Supervisor	CDP Working Group
Nicole L.	Klimas			Department of Psychiatry and Psychotherapy, LMU University Ho		Clinical data collection	CDP Working Group
Maxim	Korman			Department of Psychiatry and Psychotherapy, LMU University Ho		Clinical data collection	CDP Working Group

***First Name and Middle Initial(s)**	***Last Name**	***Suffix (eg, Jr, III)**	Academic Degrees	Institution	Location (city, state/province, country)	Role or Contribution, eg, chair, principal investigator	Group (if more than 1 Group listed in the byline) and/or Subgroup (eg, Steering Committee)
Nikolaos	Koutsouleris			Department of Psychiatry and Psychotherapy, LMU University Ho		Supervisor	CDP Working Group
Lenka	Krcmar			Department of Psychiatry and Psychotherapy, LMU University Ho		Study physician	CDP Working Group
Verena	Meisinger			Department of Psychiatry and Psychotherapy, LMU University Ho		Clinical data collection	CDP Working Group
Julian	Melcher			Department of Psychiatry and Psychotherapy, LMU University Ho		Clinical data collection	CDP Working Group
Matin	Mortazavi			Evidence-based psychiatry and psychotherapy, Faculty of Medici		Data processing	CDP Working Group
Joanna	Moussiopoulou			Department of Psychiatry and Psychotherapy, LMU University Ho		Study physician	CDP Working Group
Karin	Neumeier			Department of Psychiatry and Psychotherapy, LMU University Ho		Sample preparation an	CDP Working Group
Frank	Padberg			Department of Psychiatry and Psychotherapy, LMU University Ho		Supervisor	CDP Working Group
Boris	Papazov			Department of Psychiatry and Psychotherapy, LMU University Ho		Clinical data collection	CDP Working Group
Irina	Papazova			Department of Psychiatry, Psychotherapy, and Psychosomatics,		Clinical data collection	CDP Working Group
Sergi	Papiol			Max Planck Institute of Psychiatry, 80804 Munich, Germany		Data processing	CDP Working Group
Pauline	Pingen			Department of Psychiatry and Psychotherapy, LMU University Ho		Clinical data collection	CDP Working Group
Oliver	Pogarell			Department of Psychiatry and Psychotherapy, LMU University Ho		Supervisor	CDP Working Group
Siegfried G.	Priglinger			Department of Ophthalmology, LMU University Hospital, LMU M		Supervisor	CDP Working Group
Florian J.	Raabe			Max Planck Institute of Psychiatry, 80804 Munich, Germany		Principal Investigator	CDP Working Group
Lukas	Roell			Department of Psychiatry and Psychotherapy, LMU University Ho		Data processing	CDP Working Group
Moritz J.	Rossner			Department of Psychiatry and Psychotherapy, LMU University Ho		Supervisor	CDP Working Group
Philipp	Sämann			Max Planck Institute of Psychiatry, 80804 Munich, Germany		Supervisor	CDP Working Group
Andrea	Schmitt			Department of Psychiatry and Psychotherapy, LMU University Ho		Supervisor	CDP Working Group
Susanne	Schmölz			Department of Psychiatry and Psychotherapy, LMU University Ho		Clinical data collection	CDP Working Group
Eva C.	Schulte			Department of Psychiatry and Psychotherapy, University Hospita		Sample preparation an	CDP Working Group
Enrico	Schulz			Department of Radiology, University Hospital LMU, Ludwig-Maxi		Supervisor	CDP Working Group
Benedikt	Schworm			Department of Ophthalmology, LMU University Hospital, LMU M		Supervisor	CDP Working Group
Sophie	Seeburger			Max Planck Institute of Psychiatry, 80804 Munich, Germany		Sample preparation an	CDP Working Group
Elias	Wagner			Evidence-based psychiatry and psychotherapy, Faculty of Medici		Principal Investigator	CDP Working Group
Sven	Wichert			Department of Psychiatry and Psychotherapy, LMU University Ho		Sample preparation an	CDP Working Group
Vladislav	Yakimov			Department of Psychiatry and Psychotherapy, LMU University Ho		Study physician	CDP Working Group
Peter	Zill			Department of Psychiatry and Psychotherapy, LMU University Ho		Data processing	CDP Working Group
Michael J.	Ziller			Department of Psychiatry and Psychotherapy, University of Mün		Supervisor and Data pr	CDP Working Group
