# Supplementary material for: Bridging the Scales via Personalized Cellular Modeling and Deep Phenotyping in Schizophrenia
Source: JAMA Psychiatry. 2026 Mar 28;83(5):510–22. doi: 10.1001/jamapsychiatry.2026.0576 (PMC13033174; doi:10.1001/jamapsychiatry.2026.0576)
Supplement: Supplement 4. — Data Sharing Statement [file jamapsychiatry-e260576-s004.pdf]

Data Sharing Statement

Raabe. Bridging the Scales via Personalized Cellular Modeling and Deep Phenotyping in Schizophrenia. *JAMA Psychiatry*. Published March 28, 2026.
doi:10.1001/jamapsychiatry.2026.0576

Data
Data available: Yes
Data types: Deidentified participant data
How to access data: florian_raabe@psych.mpg.de
When available: With publication

Supporting Documents
Document types: None

Additional Information
Who can access the data: RNAseq data for this project have been deposited in the European genome phenome archive. Anonymized raw imaging, genotyping, transcriptomic and clinical data are available upon reasonable request to Florian J. Raabe (florian_raabe@psych.mpg.de), Daniel Keeser (daniel.keeser@med.uni-muenchen.de) and Michael J. Ziller (ziller@uni-muenster.de). Please note that certain subsets of patient-derived data are subject to sharing restrictions due to limited donor consent. iPSC lines used in this manuscript are available through Michael J. Ziller (ziller@uni-muenster.de) and Moritz J. Rossner (Moritz.Rossner@med.uni-muenchen.de), conditional on proper ethics approval from the requesting institution. A subset of iPSC lines is subject to sharing constraints. The code of the SPLS toolbox is publicly available: https://github.com/dpopovic30/spls_toolbox_compiled. Moreover, SVR-modelling was performed using https://github.com/neurominer-git/NeuroMiner. All relevant code including the code for gene expression imputation is available through the respective publication repository under https://github.com/zillerlab.
Types of analyses: academic purpose
Mechanisms of data availability: after approval of a proposal, conditional on proper ethics approval from the requesting institution could be necessary
Any additional restrictions: Please note that certain subsets of patient-derived data are subject to sharing restrictions due to limited donor consent.
